# Integrating contrast-enhanced ultrasound to optimize margin delineation in Mohs micrographic surgery for primary dermatofibrosarcoma protuberans: a retrospective cohort study

**DOI:** 10.3389/fonc.2026.1862377

**Published:** 2026-06-10

**Authors:** Bin Tu, Min-Hong Zou, Yan-Ying Ji, Min-Jun Lu, Yong-Zhi Xu, Jia-Lin Huang, Jun-Xue Lu, Tian Liu, Yi-Yong Hong, Xin Tang, Zhi-Kai Liao, Jian Chen, Miao-Jian Wan, Yang Xie

**Affiliations:** 1 Department of Dermatology, The Third Affiliated Hospital, Sun Yat-Sen University, Guangzhou, China; 2 Department of Ultrasound, The Third Affiliated Hospital, Sun Yat-Sen University, Guangzhou, China; 3 Department of Pathology, The Third Affiliated Hospital, Sun Yat-Sen University, Guangzhou, China; 4 Department of Dermatology, The First People's Hospital of Kashgar Region (Sun Yat-sen University Affiliated Kashgar Hospital), Kashgar, China

**Keywords:** contrast-enhanced ultrasound, dermatofibrosarcoma protuberans, high-frequency ultrasound, margin delineation, Mohs micrographic surgery, soft tissue sarcoma

## Abstract

**Introduction:**

Dermatofibrosarcoma protuberans (DFSP) is characterized by infiltrative “tentacle-like” extensions, making accurate preoperative margin delineation challenging. While high-frequency ultrasound (HFUS) visualizes subclinical spread, its ability to fully capture tumor extent remains limited. Contrast-enhanced ultrasound (CEUS), by depicting microvascular perfusion, may improve delineation, but its clinical impact on Mohs micrographic surgery (MMS) remains unclear.

**Methods:**

This retrospective cohort study included 220 primary DFSP patients treated with MMS. Preoperative margin delineation utilized HFUS-only or HFUS+CEUS. The primary outcome was extra peripheral Mohs stages. Secondary outcomes included extra deep and total stages, margin positivity, and other surgical characteristics. Multivariable Poisson regression with LASSO-based variable selection was used, alongside cohort-restriction, covariate-specification, and propensity score–matched (PSM) analyses. Risk-stratified and subgroup analyses explored effect heterogeneity.

**Results:**

HFUS+CEUS was associated with fewer extra peripheral Mohs stages than HFUS-only (adjusted incidence rate ratio [aIRR], 0.310; P = 0.049). Peripheral margin positivity was reduced from 17.4% to 5.1% (P = 0.020). These findings were consistent across sensitivity analyses and in the PSM cohort (aIRR, 0.309; P = 0.033). The relative effect was most pronounced in the high-risk stratum (IRR, 0.124; P = 0.041), although interaction testing was not statistically significant (P = 0.077). No significant differences were observed in deep or total stages, likely due to ultrasound depth limitations.

**Conclusions:**

Integrating CEUS with HFUS enhances the precision of preoperative margin delineation for DFSP, reducing extra peripheral Mohs stages. This multimodal imaging strategy represents a valuable, tissue-sparing refinement to the preoperative workflow of MMS, particularly for patients with higher baseline risk.

## 1 Introduction

Dermatofibrosarcoma protuberans (DFSP) is a rare, locally aggressive cutaneous sarcoma characterized by extensive, asymmetric subclinical spread with “tentacle-like” projections that infiltrate surrounding normal tissue (1–3). As a result, wide local excision (WLE) is associated with high rates of incomplete resection and local recurrence (4–6). Consequently, Mohs micrographic surgery (MMS) has emerged as the treatment of choice, offering superior margin control and tissue conservation (1, 7–12). However, the preoperative delineation of tumor margins remains a significant challenge. In routine practice, the initial surgical margin is often determined by clinical inspection and palpation (13, 14), an approach that is inherently subjective. The inability to accurately visualize subclinical extension preoperatively often necessitates multiple Mohs stages, increasing overall surgical burden (15, 16).

High-frequency ultrasound (HFUS) has gained prominence as a non-invasive, real-time imaging modality capable of visualizing “tentacle-like” projections of DFSP (17, 18). Although HFUS has been increasingly used in the delineation of DFSP before MMS (19, 20), it may still underestimate the true infiltration of the tumor. Contrast-enhanced ultrasound (CEUS), which can visualize microvascular perfusion, offers a theoretical advantage in defining the hypervascular borders of DFSP that may be imperceptible on HFUS (21, 22). Several recent pilot studies suggest that CEUS improves the concordance between preoperative imaging and histological dimensions, potentially refining surgical planning (21, 23).

Despite these preliminary findings, there is a paucity of comparative data quantifying the clinical benefit of adding CEUS to HFUS in the margin control of DFSP. In this retrospective DFSP study, we aimed to characterize and analyze the impact of these two preoperative delineation methods on surgical efficiency during MMS, focusing on their ability to reduce the number of extra peripheral Mohs stages required to achieve complete tumor clearance.

## 2 Materials and methods

### 2.1 Patient selection

**Figure 1:**
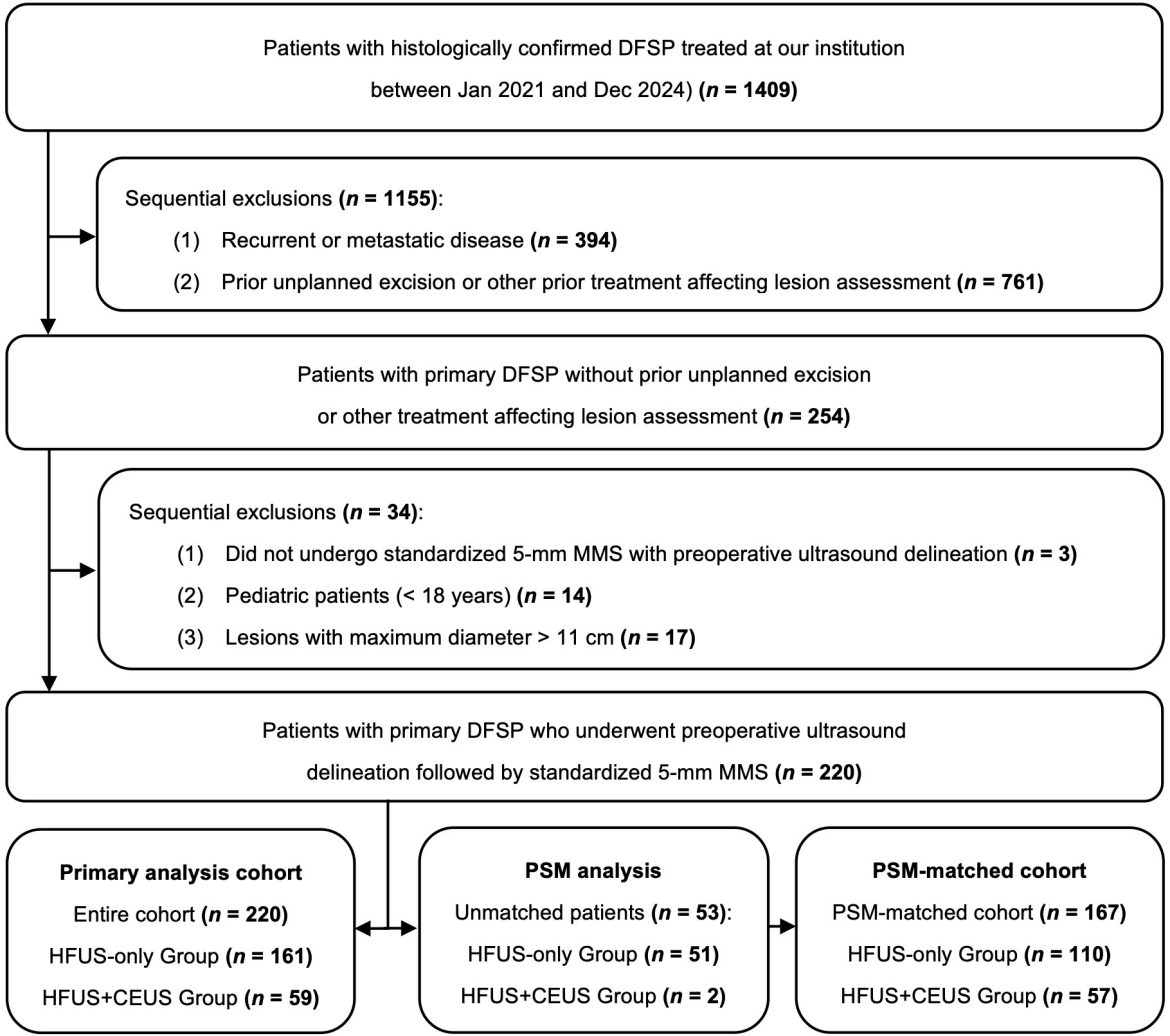
Flowchart of patient selection and propensity score matching (PSM). Pediatric patients were excluded because CEUS was not performed in individuals younger than 18 years at our institution during the study period. Lesions with a maximum diameter >11 cm were excluded because their size precluded standardized CEUS-based margin delineation within a single examination protocol. The entire cohort was used for the primary analysis, whereas the propensity score–matched cohort was used for secondary analyses to address baseline imbalance. CEUS, contrast-enhanced ultrasound; DFSP, dermatofibrosarcoma protuberans; HFUS, high-frequency ultrasound; MMS, Mohs micrographic surgery; PSM, propensity score matching.

After Institutional Review Board approval, we retrospectively reviewed 1,409 DFSP patients treated between January 1, 2021, and December 31, 2024 at the Third Affiliated Hospital of Sun Yat-Sen University, China. Patients were included if they underwent MMS with complete medical records; therefore, no missing data were present for the variables of interest. Exclusion criteria were recurrent/metastatic disease (n = 394), prior unplanned excision (n = 761), no standardized MMS with ultrasound margin delineation (n = 3), age < 18 years (n = 14), and giant tumors > 11 cm (n = 17). The final cohort comprised 220 patients (**Figure 1**).

### 2.2 Study design

This study leveraged the clinical introduction of CEUS at our institution (March 2023) to evaluate whether CEUS-assisted margin delineation reduces extra peripheral Mohs stages compared with HFUS alone. Patients were categorized into HFUS-only or HFUS+CEUS based on preoperative delineation method. From March 2023 onwards, assignment to the HFUS-only group was non-random, and detailed assignment mechanisms are provided in the **Supplementary Methods**.

### 2.3 Outcomes

The primary outcome was the number of extra peripheral Mohs stages required for tumor clearance. Secondary outcomes included extra deep and total Mohs stages, corresponding margin positivity rates (peripheral, deep, and total), reconstruction method, and local recurrence. Based on the 3D-histological mapping principles of the Mohs Tübingen technique (12), stage 1 was defined as the initial excision of lateral cylindrical and bottom disc margins. Margin positivity was defined as residual tumor in the initial margin. Extra peripheral and deep stages were defined as subsequent excisions prompted by margin positivity. Extra total stages were defined as the maximum of peripheral and deep stages.

### 2.4 Data collection and follow-up

Baseline demographic (including biological sex), clinical, and surgical characteristics were systematically extracted from electronic medical records, operative notes, and digital Mohs maps. Biological sex was defined based on phenotypic presentation at birth as recorded in the medical system; gender identity data were not available due to the retrospective nature of the study. The maximum tumor diameter was defined as the largest dimension measured clinically prior to surgery. Postoperative surgical details, including the number of peripheral and deep Mohs stages, reconstruction methods, and the deepest plane of resection, were documented.

Imaging features were retrospectively assessed from preoperative ultrasound reports using standardized criteria. US morphology was dichotomized (24): into “well-defined” (tumors with entirely circumscribed borders; **Supplementary Figure 1A**) and “ill-defined” (tumors exhibiting “finger-like” projections or other irregular boundaries; **Supplementary Figures 1B, C**). Intratumoral vascularity was evaluated using Color Doppler and classified according to the Adler semi-quantitative grading system (grades 0–1 as poor vascularity, grades 2–3 as rich vascularity). Deep fascia abutment was considered positive if the tumor’s lower margin was in direct contact with or disrupted the hyperechoic fascial line.

Pathological diagnoses were confirmed by dermatopathologists. The pathological subtype was meticulously determined based on the presence and percentage of fibrosarcomatous transformation observed in the final, fully excised surgical specimen, rather than preliminary biopsies. The presence of the *COL1A1-PDGFB* fusion gene was confirmed via fluorescence *in situ* hybridization (FISH).

Follow-up was conducted via outpatient visits or telephone interviews every 6 months for the first 2 years and annually thereafter.

### 2.5 Preoperative margin delineation and Mohs micrographic surgery

**Figure 2:**
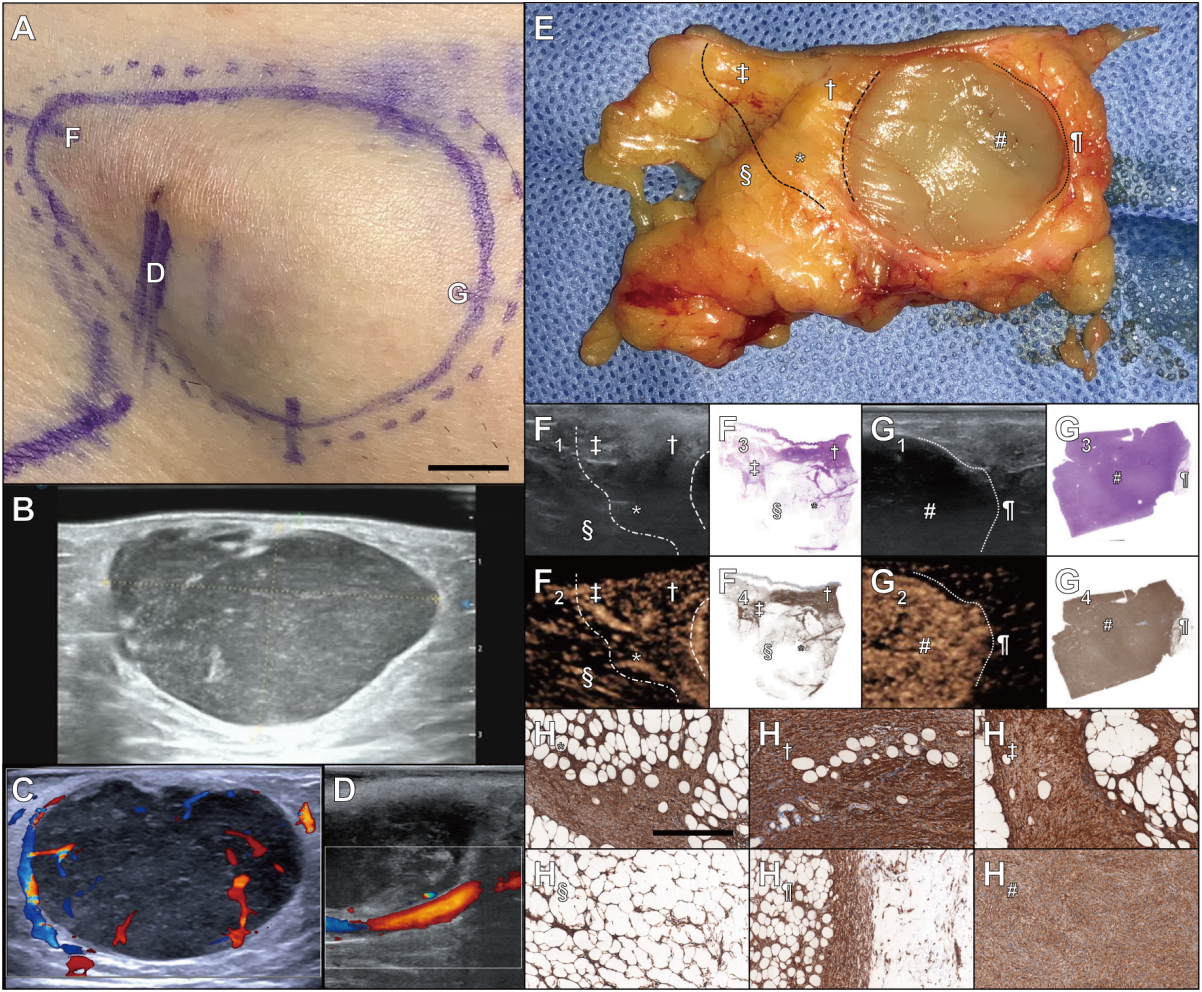
Representative multimodal correlation among clinical appearance, ultrasound imaging, macroscopic specimen, and histopathology of DFSP in a 57-year-old female (right inguinal region). Panels **(A–E, F_1_–G_4_)** are displayed at the same physical scale to allow direct spatial correlation across clinical, ultrasound, gross, and histopathological views. **(A)** Preoperative gross appearance. The solid purple line indicates the CEUS-determined tumor border, and the dashed purple line represents the planned 5-mm initial margin. F and G mark the lateral and medial borders, respectively. D indicates the superficial epigastric vascular plexus. **(B–D)** Preoperative ultrasound evaluation. Grayscale HFUS **(B)** shows the tumor mass, with Color Doppler **(C)** demonstrating intralesional vascularity and **(D)** compression of the superficial vascular plexus. **(E)** Gross pathology of the resected specimen (transverse section). # indicates the main tumor mass, and § refers to adipose tissue. ¶ marks medial border, whereas *, †, and ‡ indicate infiltrative extensions into surrounding fat. The dotted line refers to the well-defined medial margin, the dashed line indicates the apparent lateral margin, and the dash-dot line represents the CEUS-defined boundary. **(F_1_–F_4_)** Correlation of the infiltrative lateral border across HFUS **(F_1_)**, CEUS **(F_2_)**, H&E **(F_3_)**, and CD34 staining **(F_4_)**. **(G_1_–G_4_)** Correlation of the well-defined medial border across HFUS **(G_1_)**, CEUS **(G_2_)**, H&E **(G_3_)**, and CD34 staining **(G_4_)**. **(H)** High-magnification CD34-stained sections show details of the well-defined border **(H_¶_)**, the dense tumor mass **(H_#_)**, pure adipose tissue **(H_§_)**, and an admixture of tumor cells and adipose tissue (H_*_, H_†_, H_‡_). Scale bars: the scale bar in panel **(A)** represents 1 cm and applies to panels **(A–E)** and **(F_1_–G_4_)**. The scale bar in panel (H_*_) represents 400 μm and applies to all H panels.

All patients underwent preoperative HFUS to delineate tumor boundaries, with margins marked on the skin (**Figure 2A**). For the HFUS+CEUS group, this initial border was re-evaluated using CEUS, and skin markings were refined. Detailed protocols and equipment specifications were provided in the **Supplementary Methods**.

MMS was performed using a Tübingen-style margin orientation and mapping approach (12), with frozen-section evaluation. First, initial tumor debulking was performed with a 5-mm margin beyond the ultrasound-defined border (**Figure 2A**). Subsequently, thin (1–2 mm) peripheral and deep margins were excised, subdivided into anatomically oriented blocks, digitally mapped (25), and processed for frozen section analysis. Positive margins prompted targeted 2–3 mm re-excisions until clear. Finally, an additional 2–3 mm circumferential adipose safety margin was excised for permanent paraffin histology. Reconstruction was performed immediately thereafter. Post-surgery safety margin evaluation was not included in intraoperative stage counts.

### 2.6 Statistical analysis

Data were analyzed using R version 4.4.1 (R Foundation for Statistical Computing, Vienna, Austria). Baseline comparability was assessed using standardized mean differences (SMDs). Categorical variables were compared using the chi-square or Fisher’s exact test, and continuous variables using the Mann–Whitney U test.

Given the count nature of the outcome, low mean, and no evidence of overdispersion or zero inflation, Poisson regression was used as the primary model. Univariable analyses were performed to screen associations between baseline variables and the primary outcome. Delineation method, along with three clinically relevant variables (age, maximum tumor diameter, and primary site) was prespecified for multivariable analysis. Additional covariates were selected using LASSO penalization. Continuous variables were assessed for non-linearity prior to multivariable models. Details are provided in the **Supplementary Methods**. The secondary outcome (extra deep Mohs stages) was analyzed using a similar framework.

A series of sensitivity and validation analyses were conducted to assess the robustness of the findings and address potential confounding. First, the primary model was applied under alternative cohort restrictions to address time-related confounding and selection bias due to the non-random assignment of patients. Second, covariate specification models were specified using (1) clinically relevant variables, (2) LASSO-selected variables based on the λ_1-SE_ criterion, or (3) a more fully adjusted model including variables selected at λ_min_, to improve stability and reduce overfitting, particularly given the limited number of outcome events. Third, a propensity score–matched cohort was constructed using a parsimonious set of preoperative covariates selected *a priori* based on their clinical relevance to treatment allocation and surgical complexity, including age, primary site, maximum tumor diameter, ultrasound morphology, deep fascia abutment, and ultrasound depth, to mitigate baseline imbalance. In the matched cohort, outcomes were compared using Cochran–Mantel–Haenszel tests (categorical variables) and van Elteren tests (continuous variables). The primary model was then refitted in the matched sample. Finally, additional sensitivity analyses were conducted using ordinal, binary, and linear regression models as alternative outcome specifications to assess the robustness of the findings to different modeling assumptions.

To explore potential heterogeneity in the effect of delineation method, a risk-stratified analysis was performed. Patients were categorized into tertiles of predicted baseline risk derived from a logistic regression model in the HFUS-only group. Within each stratum, incidence rate ratios (IRRs) were estimated using Poisson regression models. Exploratory subgroup and interaction analyses were conducted to assess potential effect modification. Detailed model specifications are provided in the **Supplementary Methods**. A two-sided *P* value < 0.05 was considered statistically significant.

## 3 Results

### 3.1 Baseline characteristics and surgical outcomes

**Figure 3:**
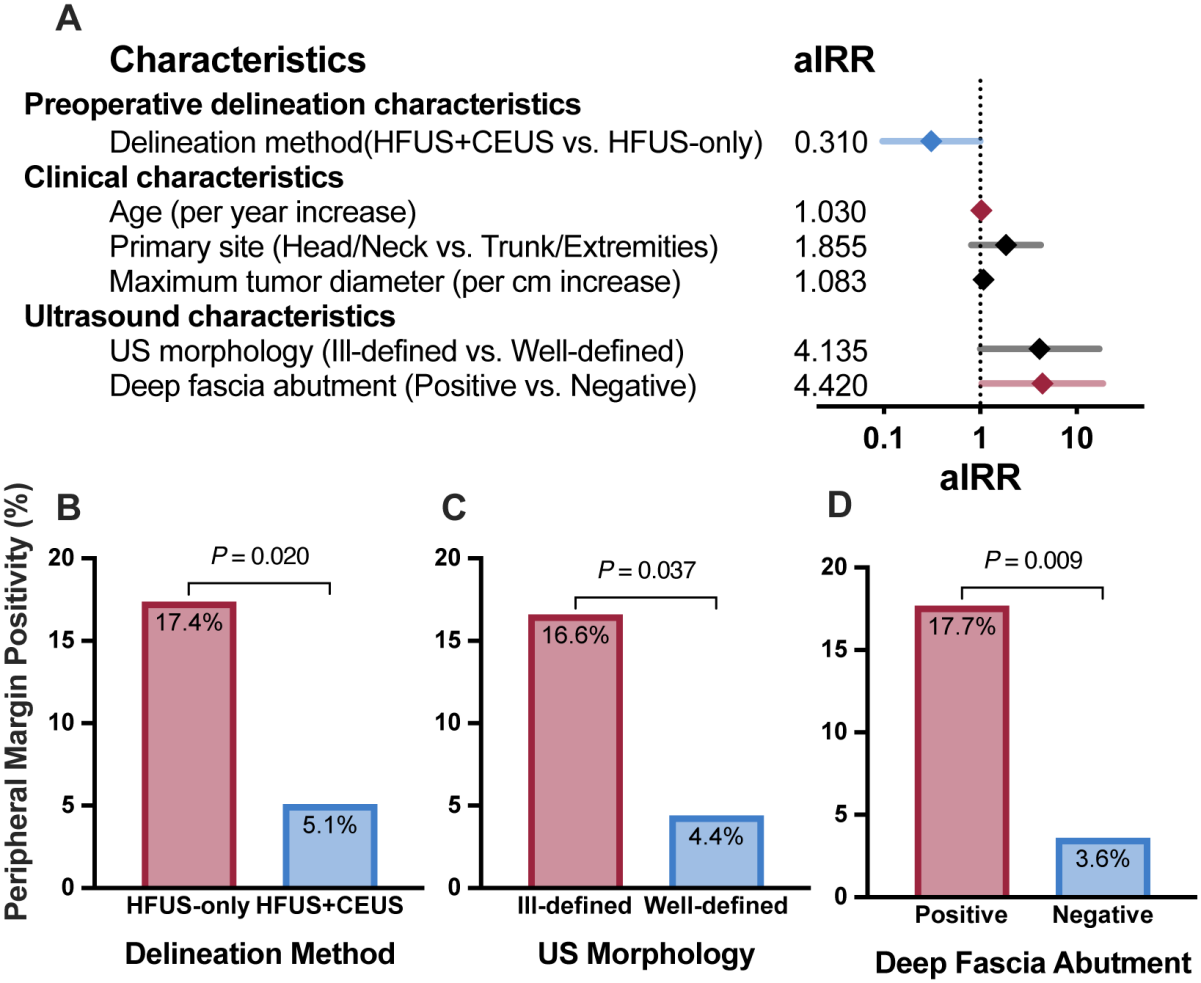
Factors associated with extra peripheral Mohs stages and corresponding peripheral margin positivity. **(A)** Forest plot showing adjusted incidence rate ratios (aIRRs) and 95% confidence intervals (CIs) from the multivariable Poisson regression model. **(B–D)** Bar charts showing peripheral margin positivity rates across key strata, stratified by delineation method **(B)**, US morphology **(C)**, and deep fascia abutment **(D)**. *P* values in panels **(B–D)** were calculated using the chi-square test. Peripheral margin positivity rates are presented for descriptive comparison. aIRR, adjusted incidence rate ratio; CEUS, contrast-enhanced ultrasound; HFUS, high-frequency ultrasound; US, ultrasound.

The final cohort comprised 220 patients, including 116 (52.7%) females and 104 (47.3%) males (**Table 1**). Most tumors were located on the trunk/extremities (94.1%), exhibited “ill-defined” US morphology (79.5%), and were *COL1A1-PDGFB* fusion-positive (95.9%). Patients in the HFUS+CEUS group were slightly older (36.7 vs. 34.6 years; SMD = 0.193) and had somewhat smaller tumors (median, 4.8 vs. 5.5 cm; SMD = 0.234). Apart from age and maximum tumor diameter, baseline characteristics were otherwise well balanced (most SMDs < 0.1). Most lesions on CEUS exhibited rapid, marked, and homogeneous hyperenhancement (**Supplementary Table 1**). Regarding surgical outcomes (**Table 2**), HFUS+CEUS was associated with a reduction in peripheral margin positivity from 17.4% to 5.1% compared with HFUS-only (*P* = 0.020; **Figure 3B**) and fewer extra peripheral Mohs stages (corresponding to mean peripheral stage counts of 1.05 vs. 1.19; *P* = 0.020). No significant differences were observed in deep stages, total stages, reconstruction method, or the deepest plane of resection (all *P* > 0.05).

**Table 1:** Baseline clinical and tumor characteristics by delineation method (HFUS-only vs HFUS+CEUS).

Characteristics	Entire cohort (n = 220)	HFUS-Only (n = 161)	HFUS+CEUS (n = 59)	SMD
Clinical characteristics				
Age, years, mean (SD)	35.2 (10.0)	34.6 (9.1)	36.7 (11.8)	0.193
Sex, No. (%)				0.067
Female	116 (52.7)	82 (50.9)	34 (57.6)	
Male	104 (47.3)	79 (49.1)	25 (42.4)	
Primary site, No. (%)				0.011
Head and neck	13 (5.9)	10 (6.2)	3 (5.1)	
Trunk and extremities	207 (94.1)	151 (93.8)	56 (94.9)	
Clinical presentation, No. (%)				0.015
Nodular/Subcutaneous	118 (53.6)	87 (54.0)	31 (52.5)	
Plaque/Atrophic	102 (46.4)	74 (46.0)	28 (47.5)	
Disease duration, months, median (IQR)	48 (24, 120)	48 (23, 120)	60 (12, 120)	0.072
Maximum tumor diameter, cm, median (IQR)	5.0 (3.7, 7.0)	5.5 (3.9, 7.0)	4.8 (3.0, 6.1)	0.234
Ultrasound characteristics				
Ultrasound morphology, No. (%)				0.022
Well-defined	45 (20.5)	32 (19.9)	13 (22.0)	
Ill-defined	175 (79.5)	129 (80.1)	46 (78.0)	
Echogenicity, No. (%)				0.012
Hyperechoic or mixed	95 (43.2)	69 (42.9)	26 (44.1)	
Hypoechoic	125 (56.8)	92 (57.1)	33 (55.9)	
Tumor depth (US), cm, median (IQR)	1.20 (0.77, 1.90)	1.21 (0.73, 1.91)	1.13 (0.84, 1.83)	0.094
Deep fascia abutment, No. (%)				0.046
Negative	56 (25.5)	39 (24.2)	17 (28.8)	
Positive	164 (74.5)	122 (75.8)	42 (71.2)	
Vascularity, No. (%)				0.051
Poor (Adler 0-1)	53 (24.1)	41 (25.5)	12 (20.3)	
Rich (Adler 2-3)	167 (75.9)	120 (74.5)	47 (79.7)	
Pathological characteristics				
Pathological subtype, No. (%)				0.022
C-DFSP	168 (76.4)	122 (75.8)	46 (78.0)	
FS-DFSP	52 (23.6)	39 (24.2)	13 (22.0)	
COL1A1-PDGFB fusion gene, No. (%)				0.010
Positive	211 (95.9)	154 (95.7)	57 (96.6)	
Negative	9 (4.1)	7 (4.3)	2 (3.4)	

Adler, a semi-quantitative classification of color Doppler blood flow; C-DFSP, classic DFSP; CEUS, contrast-enhanced ultrasound; COL1A1, collagen type I alpha 1 chain; DFSP, dermatofibrosarcoma protuberans; FS-DFSP, fibrosarcomatous DFSP; HFUS, high-frequency ultrasound; IQR, interquartile range; PDGFB, platelet-derived growth factor beta chain; SD, standard deviation; SMD, standardized mean difference; US, ultrasound.

The interquartile range (IQR) is presented as the 25th and 75th percentiles (Q1, Q3).

**Table 2:** Mohs surgical outcomes by delineation method (HFUS-only vs HFUS+CEUS).

Characteristics	Entire cohort (n = 220)	HFUS-Only (n = 161)	HFUS+CEUS (n = 59)	*P* value
Peripheral Mohs stages, Mean (SD)	1.15 (0.38)	1.19 (0.42)	1.05 (0.22)	0.020*†
Peripheral margin status, No. (%)				0.020*‡
Negative	189 (85.9)	133 (82.6)	56 (94.9)	
Positive	31 (14.1)	28 (17.4)	3 (5.1)	
Deep Mohs stages, Mean (SD)	1.30 (0.52)	1.31 (0.54)	1.27 (0.45)	0.858†
Deep margin status, No. (%)				0.975‡
Negative	160 (72.7)	117 (72.7)	43 (72.9)	
Positive	60 (27.3)	44 (27.3)	16 (27.1)	
Total Mohs stages, Mean (SD)	1.35 (0.55)	1.37 (0.58)	1.31 (0.46)	0.644†
Total margin status, No. (%)				0.801‡
Negative	150 (68.2)	109 (67.7)	41 (69.5)	
Positive	70 (31.8)	52 (32.3)	18 (30.5)	
Reconstruction method, No. (%)				0.685§
Primary closure/local flap	212 (96.4)	154 (95.7)	58 (98.3)	
Skin graft	8 (3.6)	7 (4.3)	1 (1.7)	
Deepest plane of resection, No. (%)				0.429‡
Deep fascia	136 (61.8)	97 (60.2)	39 (66.1)	
Subfascial tissue (muscle/bone)	84 (38.2)	64 (39.8)	20 (33.9)	

Total Mohs stages were defined as the maximum of peripheral and deep stages. Peripheral, deep, and total Mohs stages shown in **Table 2** refer to primary stage counts; corresponding extra stages equal stage count minus 1.

Peripheral, deep, and total Mohs stages were compared using the Mann–Whitney U test, as these variables are not normally distributed; mean (SD) values are presented for descriptive comparability with previous studies.

Peripheral, deep, and total margin status were compared using the chi-square test.

CEUS, contrast-enhanced ultrasound; DFSP, dermatofibrosarcoma protuberans; HFUS, high-frequency ultrasound; SD, standard deviation.

*Differences were considered statistically significant at P < 0.05.

†Mann-Whitney U test; ‡chi-Square test; §Fisher’s exact test. Bold values indicate statistical significance (P < 0.05).

### 3.2 Characteristics associated with extra peripheral Mohs stages

Following univariable regression and LASSO variable selection (**Supplementary Figures 2A, C**; **Supplementary Tables 2**, **3**), with no evidence of non-linearity observed for continuous variables (**Supplementary Figure 3**), multivariable Poisson regression showed that HFUS+CEUS was associated with fewer extra peripheral Mohs stages (aIRR, 0.310; *P* = 0.049), whereas older age (aIRR, 1.030; *P* = 0.025) and positive deep fascia abutment (aIRR, 4.420; *P* = 0.044) were associated with higher stage counts (**Figure 3A**; **Supplementary Table 4**). An ill-defined US morphology showed a borderline association with increased stages (aIRR, 4.135; *P* = 0.050). Descriptive comparisons also showed lower peripheral margin positivity in the HFUS+CEUS group and higher positivity in tumors with “ill-defined” US morphology or positive deep fascia abutment (**Figures 3B–D**).

### 3.3 Multimodal imaging-pathology correlation

A representative DFSP case (**Figure 2**) illustrates the margin delineation capability of CEUS. HFUS accurately delineated the well-defined medial pseudocapsule but failed to distinguish lateral infiltrative extensions and normal tissue (**Figures 2F1, G1**). In contrast, CEUS captured differential microvascular perfusion, delineating subclinical boundaries (**Figures 2F2, G2**). These CEUS-defined margins corresponded closely with histopathologic findings, particularly CD34-stained sections (**Figures 2F–H**).

### 3.4 Sensitivity, risk-stratified, and subgroup analyses

**Figure 4:**
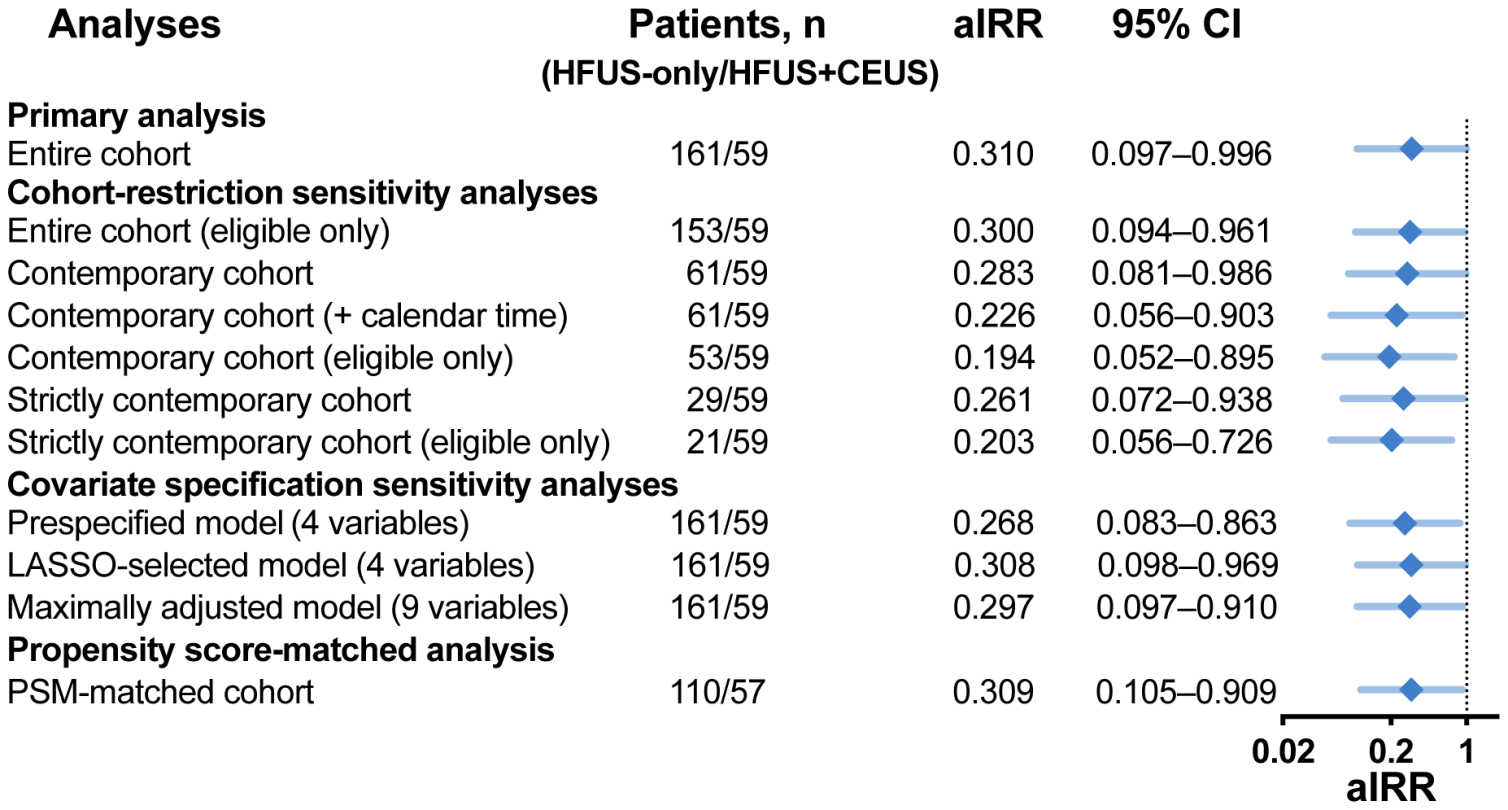
Primary and sensitivity analyses of delineation method on extra peripheral Mohs stages. Forest plot showing adjusted incidence rate ratios (aIRRs) and 95% confidence intervals (CIs) for extra peripheral Mohs stages comparing HFUS+CEUS with HFUS-only. “Eligible only” indicates exclusion of clinically ineligible HFUS-only cases, defined as lesions deemed too small for CEUS despite availability. The contemporary cohort included patients treated during the CEUS implementation period, whereas the strictly contemporary cohort included those treated during periods of confirmed CEUS availability, excluding downtime intervals. Calendar-time adjustment was performed by additionally including an ordinal per-6-month time variable in the multivariable model. Covariate-specification sensitivity analyses included prespecified, LASSO-selected (λ_1-SE_), and maximally adjusted (λ_min_) models. The PSM-matched cohort represents analyses conducted in the propensity score–matched sample to further address baseline imbalance. All estimates were derived from multivariable Poisson regression models. Values below 1 indicate fewer extra peripheral Mohs stages in the HFUS+CEUS group. aIRR, adjusted incidence rate ratio; CI, confidence interval; CEUS, contrast-enhanced ultrasound; HFUS, high-frequency ultrasound; PSM, propensity score matching.

No statistically detectable calendar-time trend was observed (**Supplementary Table 5**). Sensitivity analyses under alternative cohort restrictions yielded consistent results (**Figure 4**; **Supplementary Table 6**), with good covariate balance observed across these cohorts (most SMDs < 0.1) (**Supplementary Table 7**). In covariate specification sensitivity analyses, two more parsimonious models (4 variables) and a more fully adjusted model (9 variables) demonstrated similar results (**Figure 4**; **Supplementary Table 8**).

PSM achieved good covariate balance, with all SMDs < 0.1 (**Supplementary Figure 4**; **Supplementary Table 9**). After matching, 57 patients in the HFUS+CEUS group were matched to 110 patients in the HFUS-only group. In the matched cohort, HFUS+CEUS was associated with a lower peripheral margin positivity rate and fewer extra peripheral Mohs stages compared with HFUS-only (**Supplementary Table 10**). Multivariable Poisson regression further confirmed this association (aIRR, 0.309; *P* = 0.033) (**Figure 4**; **Supplementary Table 8**). Sensitivity analyses using alternative models showed consistent findings.

**Figure 5:**
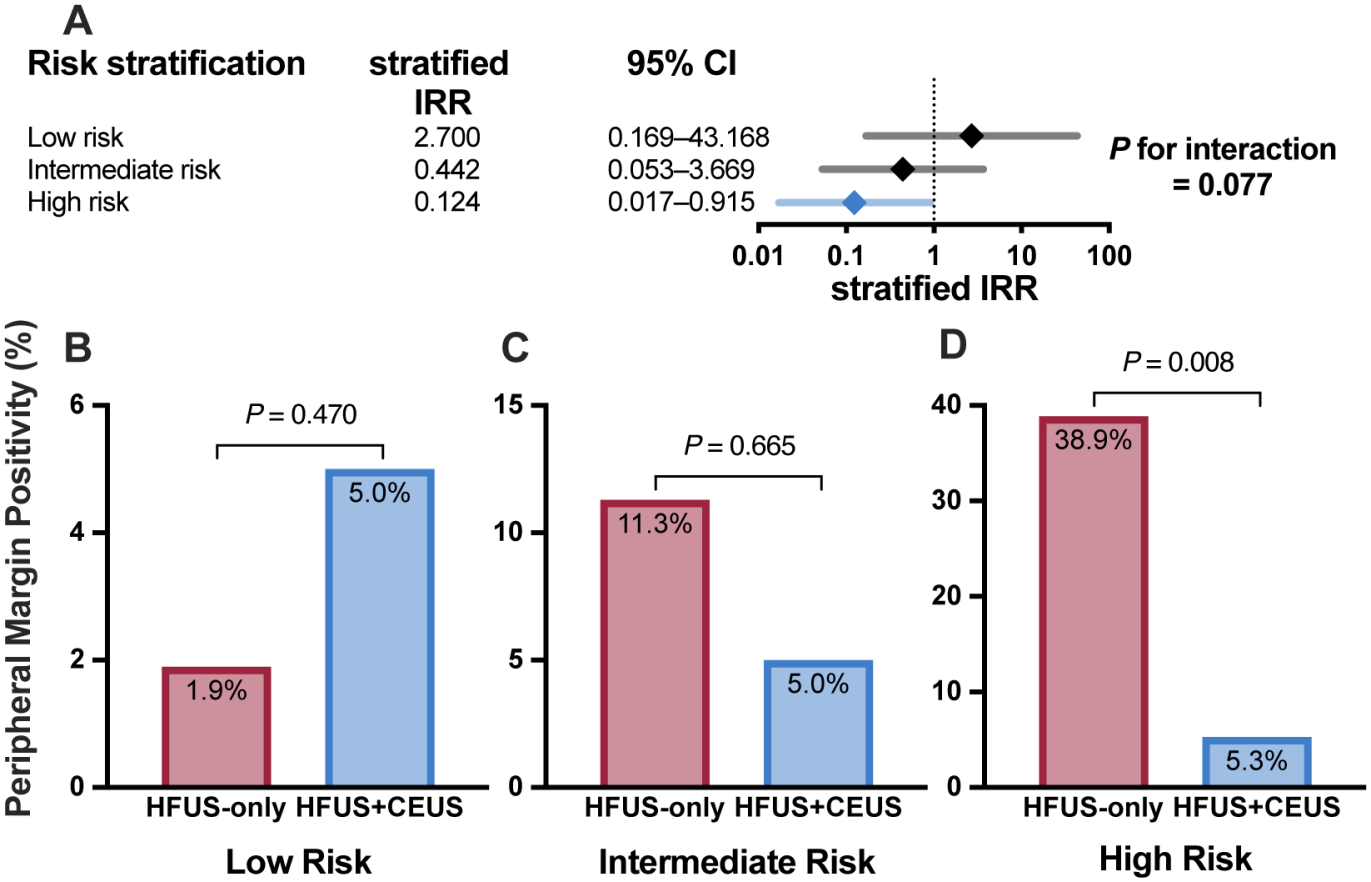
Risk-stratified analysis of delineation method for extra peripheral Mohs stages. **(A)** Forest plot showing incidence rate ratios (IRRs) and 95% confidence intervals (CIs) comparing HFUS+CEUS with HFUS-only across risk strata defined by tertiles of predicted baseline risk derived from the HFUS-only group. IRRs were estimated using Poisson regression models within each stratum. Interaction between delineation method and risk strata was formally tested in the full cohort. **(B–D)** Bar charts showing peripheral margin positivity rates within each risk stratum: low risk **(B)**, intermediate risk **(C)**, and high risk **(D)**. Peripheral margin positivity rates are presented for descriptive comparison. *P* values in panels **(B–D)** were calculated using Fisher’s exact test. IRR, incidence rate ratio; CI, confidence interval; CEUS, contrast-enhanced ultrasound; HFUS, high-frequency ultrasound.

Using a baseline risk model derived from the HFUS-only group (**Supplementary Table 11**), patients were stratified into risk tertiles (**Supplementary Table 12**). The relative effect of HFUS+CEUS was most pronounced in the high-risk stratum (IRR, 0.124; *P* = 0.041; **Figure 5**). HFUS+CEUS was associated with a reduction in the peripheral margin positivity rate from 38.9% to 5.3% compared with HFUS-only (*P* = 0.008; **Figure 5D**), although formal interaction testing did not reach statistical significance (*P* for interaction = 0.077).

Subgroup analyses showed consistent associations of HFUS+CEUS across strata, with no significant interaction effects (**Supplementary Table 13**). Associations among key characteristics were further explored (**Supplementary Table 14**).

### 3.5 Factors associated with deep Mohs stages

A parallel analytical framework was applied to evaluate extra deep Mohs stages (**Supplementary Figures 2B, D**; **Supplementary Tables 15**, **16**). Multivariable Poisson regression showed that head and neck location (aIRR, 2.828; *P* < 0.001), “ill-defined” US morphology (aIRR, 3.312; *P* = 0.025), and positive deep fascia abutment (aIRR, 3.119; *P* = 0.014) were independent risk factors (**Supplementary Figure 5A**; **Supplementary Table 17**).

### 3.6 Follow-up and clinical outcomes

Postoperatively, the paraffin safety margins in adipose tissue were found to be positive in 6 patients: 5 (3.1%) in the HFUS-only group and 1 (1.7%) in the HFUS+CEUS group. All patients underwent targeted re-excision to achieve complete clearance. No local recurrences or distant metastases have been observed after a median follow-up of 24 months (range, 11–50 months).

## 4 Discussion

Given the rarity of DFSP, this represents, to our knowledge, the largest retrospective study evaluating multimodal ultrasound for preoperative margin delineation of MMS. Our study demonstrated that HFUS+CEUS was associated with fewer extra peripheral Mohs stages compared with HFUS-only, accompanied by lower peripheral margin positivity. Although this retrospective, non-randomized design remains susceptible to residual confounding and temporal bias, consistent findings across multiple analytical strategies and sensitivity analyses support the robustness of the association.

In our experience, HFUS reliably identifies hypoechoic “tentacle-like” projections (**Supplementary Figure 1B**) histologically densely packed with tumor cells (26), but struggles with mixed-echogenic borders (**Figure 2F_1_**; **Supplementary Figure 1C**). Histologically, tissue with more tumor cells and fewer adipocytes is slightly hyperechoic (**Figure 2H_†_**), while tissue with fewer tumor cells is relatively hypoechoic (Figure 2H_*_). In contrast, CEUS may help delineate these ambiguous boundaries by visualizing real-time microvascular perfusion (**Figure 2F_2_**), thereby providing a plausible mechanistic explanation for the accuracy of margin delineation.

Historically, most pivotal studies evaluating MMS for DFSP have reported a total stage count (27–29), as conventional MMS blurs the spatial distinction between peripheral and deep margins, which were separately analyzed in our study using a Tübingen-style margin orientation approach. While DFSP exhibits unpredictable horizontal extension, identifying its deep vertical boundaries ultrasonographically remains challenging. Neither HFUS nor CEUS can reliably delineate deep tumor margins due to inherent acoustic attenuation at greater depths and the confounding effect of posterior acoustic enhancement artifacts frequently observed behind these lesions. This obscures the true deep histological margins. Consequently, preoperative margin delineation primarily optimizes the peripheral margin assessment. Evaluating total Mohs stages as a single outcome would dilute the measurable benefit of CEUS in peripheral margin control, whereas separating margins provides a mechanism-aligned assessment of peripheral tumor spread.

While the absolute reduction appears modest (0.14 stages), its clinical significance is substantial. The integration of CEUS was associated with a marked decrease in peripheral margin positivity (from 17.4% to 5.1% overall, and from 38.9% to 5.3% in the high-risk stratum), resulting in a high single-stage clearance rate of approximately 95%. Achieving ~95% single-stage clearance represents substantial optimization of surgical workflow. The relatively low total Mohs stages counts in our cohort (1.37 in HFUS-only and 1.31 in HFUS+CEUS) compared with recent large-scale series (1, 29–31) (1.47–1.90) may reflect the benefit of this margin-assessment approach. However, such historical cross-study comparisons must be interpreted with caution. While not explicitly measured in this study, reducing the number of extra peripheral Mohs stages theoretically helps to decrease overall operative time, alleviate the laboratory burden of processing subsequent frozen sections, and potentially mitigate intraoperative patient anxiety.

The clinical benefits of CEUS must be carefully weighed against its logistical demands and resource utilization, including intravenous contrast administration, extended procedural time, and highly specialized sonographers. These constraints were reflected in our data: during periods of CEUS availability, 16 patients missed the modality simply due to experienced CEUS sonographer unavailability, and 8 were deemed clinically unsuitable due to small tumor size. Given these limitations, adopting CEUS as a blanket routine may not be cost-effective or feasible in settings where CEUS is not readily available. Instead, our findings support a targeted, risk-stratified approach. This is supported by the observed gradient in effect estimates across risk strata, with the lowest aIRR (0.124; **Figure 5**) in the high-risk group, suggesting a potentially greater relative benefit in higher-risk patients, particularly those with “ill-defined” US morphology or deep fascia abutment, along with other features contributing to higher baseline risk, such as larger tumor size, older age, and head and neck location. However, formal interaction testing did not reach statistical significance, and these findings should be considered hypothesis-generating.

Beyond the imaging modality, multivariable analyses identified key characteristics influencing surgical difficulty. First, “ill-defined” US morphology was a key determinant of complexity, reflecting an infiltrative growth pattern. Second, deep fascia abutment was associated with horizontal and vertical infiltration, as tumor infiltration may extend into muscle and spread along fascial planes after approaching the deep fascia (32). Additionally, head and neck tumors necessitated more deep stages, consistent with previous studies (29), which may reflect regional anatomical complexity (33). In line with prior reports (29, 34), larger tumor diameter showed borderline association in univariable analysis but not after adjustment, which may represent confounding by fascia abutment (**Supplementary Table 14**). Older age was also associated with increased peripheral stages, possibly reflecting more complex tumor characteristics in older patients, although age itself is unlikely to be a direct causal factor. Although fibrosarcomatous DFSP (FS-DFSP) is typically considered more aggressive, it was not retained after LASSO penalization. FS-DFSP correlated with larger tumor size, deep fascia abutment, and a more “well-defined” ultrasound morphology (**Supplementary Table 14**). This pattern may partly explain why FS-DFSP was not independently retained, although the underlying biological interpretation remains uncertain.

Currently, no guidelines recommend routine imaging-based margin delineation for DFSP prior to MMS (13, 14). While visual inspection and palpation are inherently operator-dependent and subjective (35, 36), HFUS provides an imaging-based alternative (19, 20), but requires experience for interpreting “ill-defined” margins (**Supplementary Figures 1B, C**). CEUS offers clearer contrast between tumor perfusion and surrounding tissue, improving delineation objectivity. Therefore, our findings suggest a potential role for integrating CEUS into the preoperative margin delineation workflow, particularly in patients at higher baseline risk. If unavailable, HFUS remains useful, especially for “well-defined” tumors where a more conservative or tailored resection margin may be appropriate. Notably, this tailored approach can be applied asymmetrically even within a single lesion (**Figure 2**), with narrower margins for “well-defined” borders (**Figure 2G**) and wider margins for “ill-defined” extensions (**Figure 2F**). This asymmetrical refinement suggests that CEUS improves the true morphological accuracy of margin delineation, rather than simply leading to a uniformly larger initial excision.

Furthermore, to achieve a comprehensive preoperative imaging strategy, the blind spots of ultrasound must be recognized. Given the inherent limitations of ultrasound in visualizing deep tissue, advanced imaging modalities like Magnetic Resonance Imaging (MRI) and Computed Tomography (CT) remain highly beneficial for comprehensive surgical planning. Unlike ultrasound, MRI is not constrained by acoustic attenuation and provides exceptional soft-tissue contrast, making it the optimal modality for capturing deep vertical tumor growth and identifying fascial or muscular intercalation. Additionally, CT serves as a critical complementary tool for evaluating potential cortical bone erosion. While HFUS and CEUS offer high-resolution, real-time mapping of horizontal subclinical extensions in the superficial subcutaneous fat, MRI and CT serve as essential complementary tools for deep margin assessment, particularly for tumors that are massive or exhibit potential invasion into deep critical structures.

No local recurrences or distant metastases were observed during the follow-up period (median, 24 months). This finding is compatible with short-term oncological safety but should be interpreted cautiously given the limited follow-up duration. Routine evaluation of paraffin safety margins may have contributed by mitigating the known limitations of frozen-section assessment in adipose tissue, such as freeze artifacts and fat dropout (37–39).

Our study has several limitations. First, this was a retrospective single-center study with non-randomized allocation. Residual selection and temporal biases cannot be excluded despite consistent sensitivity analyses. Second, follow-up remains short for a tumor with late recurrence potential (40). Third, HFUS and CEUS interpretation remains operator-dependent (17). Fourth, technical limitations restrict use in very large tumors. Finally, key clinical outcomes such as operative time and patient-reported experience, and precise quantitative measurements of final surgical defect sizes were not measured, limiting full clinical impact assessment.

In conclusion, integrating CEUS with HFUS was associated with improved preoperative margin delineation and better peripheral margin control, as reflected by a reduced need for extra peripheral Mohs stages and a higher likelihood of single-stage clearance. It may represent a valuable adjunct in selected cases, particularly for optimizing peripheral margin control in tumors with an “ill-defined” morphology. Even when CEUS is unavailable, routine HFUS assessment remains useful to support a more tailored and potentially asymmetric initial resection strategy.

## Data Availability

The original contributions presented in the study are included in the article/**Supplementary Material**. Further inquiries can be directed to the corresponding author/s.

## References

[1] Durack A Gran S Gardiner MD Jain A Craythorne E Proby CM . A 10‐year review of surgical management of dermatofibrosarcoma protuberans. Br J Dermatol. (2021) 184:731–9. doi: 10.1111/bjd.19346 32599647

[2] Hoesly PM Lowe GC Lohse CM Brewer JD Lehman JS . Prognostic impact of fibrosarcomatous transformation in dermatofibrosarcoma protuberans: A cohort study. J Am Acad Dermatol. (2015) 72:419–25. doi: 10.1016/j.jaad.2014.11.020 25582537

[3] Kus KJB Nassief G Orlowski T Poblete-Lopez C Lucas J Meine JG . Pediatric dermatofibrosarcoma protuberans: Outcomes from a single institution. J Am Acad Dermatol. (2026). doi: 10.1016/j.jaad.2026.02.050 41713584

[4] Serra-Guillén C Llombart B Nagore E Guillén C Requena C Kindem S . Histologic features associated with deep invasion in dermatofibrosarcoma protuberans. Actas Dermosifiliogr. (2016) 107:414–20. doi: 10.1016/j.ad.2016.02.001 26944448

[5] Sanabria A Pinillos P Chiesa-Estomba C Guntinas-Lichius O Kowalski LP Mäkitie AA . Comparing Mohs micrographic surgery and wide local excision in the management of head and neck dermatofibrosarcoma protuberans: A scoping review. J Dermatol Treat. (2024) 35:2295816. doi: 10.1080/09546634.2023.2295816 38146660

[6] Zhou MH Stirrat TP Alam M Bordeaux JS Brewer JD Minkis K . Dermatofibrosarcoma protuberans: A clinical review of diagnosis and management. J Am Acad Dermatol. (2026). doi: 10.1016/j.jaad.2026.02.072 41740895

[7] Gloster HM Harris KR Roenigk RK . A comparison between Mohs micrographic surgery and wide surgical excision for the treatment of dermatofibrosarcoma protuberans. J Am Acad Dermatol. (1996) 35:82–7. doi: 10.1016/s0190-9622(96)90597-6 8682970

[8] Veronese F Boggio P Tiberio R Gattoni M Fava P Caliendo V . Wide local excision vs. Mohs Tübingen technique in the treatment of dermatofibrosarcoma protuberans: A two-centre retrospective study and literature review. J Eur Acad Dermatol Venereol. (2017) 31:2069–76. doi: 10.1111/jdv.14378 28573714

[9] Gualdi G La Rosa G Di Buduo A Paradisi A Soglia S Calzavara‐Pinton P . Conventional surgery compared with formalin‐fixed tissue Mohs surgery (slow Mohs) for DFSP: A comparative analysis of 83 cases. J Eur Acad Dermatol Venereol. (2023) 37:e1393–5. doi: 10.1111/jdv.19336 37458274

[10] Remiszewski P Pisklak A Filipek K Spałek MJ Szumera-Ciećkiewicz A Szostakowski B . Dermatofibrosarcoma protuberans (DFSP): Current treatments and clinical trials. Curr Treat Options Oncol. (2025) 26:967–89. doi: 10.1007/s11864-025-01348-y 41042442 PMC12552357

[11] Snow SN Gordon EM Larson PO Bagheri MM Bentz ML Sable DB . Dermatofibrosarcoma protuberans: A report on 29 patients treated by Mohs micrographic surgery with long-term follow-up and review of the literature. Cancer. (2004) 101:28–38. doi: 10.1002/cncr.20316 15221986

[12] Cammarata E Esposto E Veronese F Airoldi C Zavattaro E Boggio P . Safety margins for dermatofibrosarcoma protuberans: A comparison between wide local excision and Mohs Tubingen technique. Eur J Dermatol. (2020) 30:289–93. doi: 10.1684/ejd.2020.3771 32576543

[13] Bordeaux J Blitzblau R Aasi SZ Alam M Amini A Bibee K . Dermatofibrosarcoma protuberans, version 1.2025, NCCN clinical practice guidelines in oncology. J Natl Compr Canc Netw. (2025) 23:e250001. doi: 10.6004/jnccn.2025.0001 39819674

[14] Saiag P Lebbe C Brochez L Emile J-F Forsea AM Harwood C . Diagnosis and treatment of dermatofibrosarcoma protuberans. European interdisciplinary guideline – update 2024. Eur J Cancer. (2025) 218:115265. doi: 10.1016/j.ejca.2025.115265 39904126

[15] Serra-Guillén C Llombart B Nagore E Guillén C Requena C Traves V . Mohs micrographic surgery in dermatofibrosarcoma protuberans allows tumour clearance with smaller margins and greater preservation of healthy tissue compared with conventional surgery: A study of 74 primary cases. Br J Dermatol. (2015) 172:1303–7. doi: 10.1111/bjd.13417 25244003

[16] Sabir MH Javed MN Owais M Talha M . Reframing robotics in Mohs surgery for rare cutaneous sarcomas: Conceptual promise and clinical realities in precision oncology. J Robot Surg. (2025) 19:467. doi: 10.1007/s11701-025-02643-4 40782288

[17] Algarin YA Pulumati A Tan J Zeitouni NC . Advances in non‐invasive imaging for dermatofibrosarcoma protuberans: A review. Australas J Dermatol. (2024) 65:610–20. doi: 10.1111/ajd.14366 39361531 PMC11629142

[18] Diago A Llombart B Serra‐Guillen C Arana E Guillén C Requena C . Usefulness of ultrasound in dermatofibrosarcoma protuberans and correlation with histopathological findings: A series of 30 cases. Skin Res Technol. (2021) 27:701–8. doi: 10.1111/srt.13003 33455037

[19] Liu Y Huang K Chen M Zhao S He Z Lu L . High-frequency ultrasound-assisted Mohs micrographic surgery for the treatment of dermatofibrosarcoma protuberans. J Plast Reconstr Aesthet Surg. (2024) 96:186–95. doi: 10.1016/j.bjps.2024.07.013 39094373

[20] Liu Y Zhao S Wei T . High‐frequency ultrasonography combined with Mohs micrographic surgery for treating recurrent DFSP. J Eur Acad Dermatol Venereol. (2025) 39:e983–6. doi: 10.1111/jdv.20722 40298455

[21] Gong X Li J Ding A Chen J Tao X Xiong P . Multimodal ultrasound for preoperative evaluation of dermatofibrosarcoma protuberans: A series of 40 cases. BMC Cancer. (2022) 22:1137. doi: 10.1186/s12885-022-10211-4 36335314 PMC9637320

[22] Ma C Sun Y Yang X Zhang Q Zhang C Cui L . Improving precision of resection by pre-surgery inspections with contrast-enhanced ultrasound for dermatofibrosarcoma protuberans: CEUS for DFSP surgical treatment. Dermatol Ther. (2016) 29:473–5. doi: 10.1111/dth.12401 27572421

[23] Gong X Li J Ding A Zuo J Rao Y Chen J . Conventional and contrast-enhanced ultrasound in the differential diagnosis of recurrent dermatofibrosarcoma protuberans and postoperative scar. BMC Cancer. (2024) 24:285. doi: 10.1186/s12885-024-11991-7 38438997 PMC10910735

[24] Zou M-H Huang Q Yang T Jiang Y Zhang L Xie Y . Role of ultrasound in the diagnosis of primary and recurrent dermatofibrosarcoma protuberans. BMC Cancer. (2021) 21:909. doi: 10.1186/s12885-021-08476-2 34376150 PMC8356448

[25] Xie Y Bai J Han L Xia Y Li L Wan M . A novel way of mapping in Mohs micrographic surgery. J Am Acad Dermatol. (2021) 85:e145–6. doi: 10.1016/j.jaad.2019.03.019 30885761

[26] Shin YR Kim JY Sung MS Jung JH . Sonographic findings of dermatofibrosarcoma protuberans with pathologic correlation. J Ultrasound Med. (2008) 27:269–74. doi: 10.7863/jum.2008.27.2.269 18204018

[27] Ratner D Thomas CO Johnson TM Sondak VK Hamilton TA Nelson BR . Mohs micrographic surgery for the treatment of dermatofibrosarcoma protuberans. J Am Acad Dermatol. (1997) 37:600–13. doi: 10.1016/S0190-9622(97)70179-8 9344201

[28] Garcia C Clark RE Buchanan M . Dermatofibrosarcoma protuberans. Int J Dermatol. (1996) 35:867–71. doi: 10.1111/j.1365-4362.1996.tb05053.x 8970843

[29] Serra-Guillén C Llombart B Nagore E Guillén C Sanmartín O . Determination of margins for tumor clearance in dermatofibrosarcoma protuberans: A single-center study of 222 cases treated with modified Mohs surgery. Dermatol Surg. (2022) 48:51–6. doi: 10.1097/DSS.0000000000003269 34743125

[30] Lee SH Oh Y Nam KA Oh B Roh MR Chung KY . Mohs micrographic surgery for dermatofibrosarcoma protuberans: Comparison of frozen and paraffin techniques. J Eur Acad Dermatol Venereol. (2018) 32:2171–7. doi: 10.1111/jdv.15201 30067886

[31] Lowe GC Onajin O Baum CL Otley CC Arpey CJ Roenigk RK . A comparison of Mohs micrographic surgery and wide local excision for treatment of dermatofibrosarcoma protuberans with long-term follow-up: The Mayo Clinic experience. Dermatol Surg. (2017) 43:98–106. doi: 10.1097/DSS.0000000000000910 27749444

[32] Foshee JP Trofymenko O Zeitouni NC . Surgical and functional considerations of dermatofibrosarcoma protuberans involving facial nerve danger zones. J Clin Aesthetic Dermatol. (2019) 12:39–43. PMC7002043 32038764

[33] Llombart B Serra-Guillén C Rubio L Nagore E Requena C Traves V . Subcutaneous dermatofibrosarcoma protuberans, a rare subtype with predilection for the head: A retrospective series of 18 cases. J Am Acad Dermatol. (2017) 77:503–511.e1. doi: 10.1016/j.jaad.2017.02.046 28420485

[34] Parker TL Zitelli JA . Surgical margins for excision of dermatofibrosarcoma protuberans. J Am Acad Dermatol. (1995) 32:233–6. doi: 10.1016/0190-9622(95)90132-9 7829708

[35] Kim BJ Kim H Jin US Minn KW Chang H . Wide local excision for dermatofibrosarcoma protuberans: A single-center series of 90 patients. BioMed Res Int. (2015) 2015:642549. doi: 10.1155/2015/642549 26688814 PMC4673335

[36] Wortsman X . Ultrasound in dermatology: Why, how, and when? Semin Ultrasound CT MR. (2013) 34:177–95. doi: 10.1053/j.sult.2012.10.001 23768885

[37] Orchard GE Shams M . Dermatofibrosarcoma protuberans: Dealing with slow Mohs procedures employing formalin-fixed, paraffin wax-embedded tissue in a busy diagnostic laboratory. Br J BioMed Sci. (2012) 69:56–61. doi: 10.1080/09674845.2012.12002437 22872928

[38] Shoufani R Berl A Shir-az O Kidron D Mann D Castel N . Overcoming Mohs limitations in treating DFSP: Retrospective analysis of a novel excision technique. Life. (2025) 15:1025. doi: 10.3390/life15071025 40724527 PMC12300959

[39] Nieto-Benito LM Ciudad-Blanco C Sanmartin-Jimenez O Garces JR Rodríguez-Prieto MA Vilarrasa E . Mohs micrographic surgery in dermatofibrosarcoma protuberans: Rate and risk factors for recurrence in a prospective cohort study from the Spanish Registry of Mohs Surgery (REGESMOHS) and review of the literature. Exp Dermatol. (2021) 30:717–22. doi: 10.1111/exd.14291 33523531

[40] Foroozan M Sei J-F Amini M Beauchet A Saiag P . Efficacy of Mohs micrographic surgery for the treatment of dermatofibrosarcoma protuberans: Systematic review. Arch Dermatol. (2012) 148:1055–63. doi: 10.1001/archdermatol.2012.1440 22986859

