# Supplementary material for: Integrating contrast-enhanced ultrasound to optimize margin delineation in Mohs micrographic surgery for primary dermatofibrosarcoma protuberans: a retrospective cohort study
Source: Front Oncol. 2026 Jun 10;16:1862377. doi: 10.3389/fonc.2026.1862377 (PMC13290673; doi:10.3389/fonc.2026.1862377)
Supplement: Supplementary file 1 [file DataSheet1.pdf]

Supplementary Methods

1. Detailed Information about Nonrandom Assignment

Contrast-enhanced ultrasound (CEUS) was performed between March 1, 2023 and September 20, 2024. Due to equipment maintenance, CEUS services were temporarily unavailable during two periods: July 1 to August 15, 2023, and April 16 to August 18, 2024.

During the periods of CEUS availability, a total of 88 patients were evaluated. Of these, 59 patients received both high-frequency ultrasound (HFUS) and CEUS, while 29 patients underwent HFUS alone. CEUS was not used in these 29 cases for the following reasons: unavailability of a specialized CEUS sonographer (n = 16); clinical assessment by the surgeon that the lesion was too small to warrant CEUS (n = 8); and patient refusal due to concerns regarding potential adverse reactions, such as allergies (n = 5). These nonrandom assignment mechanisms motivated the predefined sensitivity analyses restricted to contemporary, strictly contemporary, and clinically eligible cohorts.

The 8 patients were considered as "clinically ineligible cases" (tumors deemed too small to warrant CEUS), and all the 29 patients were classified as "HFUS-only cases from CEUS-available periods" for cohort-restriction sensitivity analyses.

2. Multimodal Ultrasound Protocols and Equipment Specifications

2.1 High-Frequency Ultrasound (HFUS): All patients underwent preoperative HFUS using an XHD ultra-high-frequency ultrasound system (Wisonic Medical Technology Co., Ltd., Shenzhen, China) equipped with variable-frequency linear-array probes (nominal frequencies of 15 and 30 MHz; typically operated at approximately 16 MHz in clinical practice). Continuous multiplanar scans were performed by an experienced sonographer to delineate tumor boundaries. The suspected margin was marked on the skin with a sterile surgical pen.

2.2 Contrast-Enhanced Ultrasound (CEUS): For patients in the HFUS+CEUS group, the initial HFUS border was re-evaluated using three CEUS platforms: EPIQ 7 ultrasound system (Philips Medical Systems, Bothell, WA, USA), ACUSON Sequoia ultrasound system (Siemens Medical Solutions USA, Inc., Mountain View, CA, USA), and P80 ultrasound system (SonoScape Medical Corp., Shenzhen, China). The contrast agent SonoVue (Bracco Imaging S.p.A., Milan, Italy) was prepared and administered intravenously according to the manufacturer's instructions. After injection, the dynamic microvascular perfusion of the tumor was continuously observed, and the initial skin markings were subsequently refined based on the real-time hyperenhanced boundary.

3. Statistical model specifications and parameter settings

3.1 R implementation and packages: All statistical analyses were performed using R version 4.4.1 (R Foundation for Statistical Computing, Vienna, Austria). Data management and preprocessing were conducted using *base R* and *tidyverse* packages. Key analyses included LASSO penalization (*glmnet*), propensity score matching

(*MatchIt*), covariate balance assessment (*cobalt*), and robust variance estimation (*sandwich* and *lmtest*). Additional analyses were conducted using standard R functions and relevant packages as appropriate.

3.2 LASSO penalization and primary regression model:

The primary outcome (extra peripheral Mohs stages) was analyzed using Poisson regression, given its count nature, low mean, and absence of substantial overdispersion or zero inflation. Candidate baseline variables were entered into a LASSO-penalized Poisson regression model. To improve selection stability, 10-fold cross-validation was repeated 50 times with different random seeds, and variables selected at $\lambda_{1\text{-SE}}$ in more than 40% of iterations were retained. Delineation method, along with three clinically relevant variables (age, maximum tumor diameter, and primary site), was prespecified and retained in the model based on their potential role as confounders and prior evidence of association with surgical complexity. Variables identified by LASSO were then added to construct the final multivariable Poisson regression model. The functional form of continuous variables was assessed using restricted cubic splines (RCS). Variables were modeled as linear terms if no evidence of nonlinearity was detected. This variable-selection strategy was intended to balance clinical interpretability, model stability, and overfitting control in an observational association analysis. In the unmatched cohort, inference for regression models was primarily based on Wald tests using heteroskedasticity-robust variance estimators, and 95% confidence intervals were calculated from the corresponding robust standard errors.

The secondary outcome (extra deep Mohs stages) was analyzed using an analogous modeling strategy. Specifically, delineation method and prespecified clinically relevant variables (age, maximum tumor diameter, and primary site) were retained a priori, and additional covariates were selected using LASSO with a lower selection-frequency threshold (>10% at $\lambda_{1\text{-SE}}$) to account for the higher event burden. Variables identified by LASSO were then combined with the prespecified variables to construct the final multivariable Poisson regression model.

3.3 Secondary validation analyses:

A series of prespecified sensitivity analyses were conducted to evaluate the robustness of the primary findings and assess potential model dependence.

3.3.1 Cohort-restriction analyses: To minimize selection bias and potential time-related confounding, the primary model was refitted in sensitivity analyses under alternative cohort restrictions.

3.3.2 Covariate specification analyses:

To reduce overfitting and improve stability of estimates, the primary model was refitted in sensitivity analyses using three alternative specifications: a prespecified model, including the three prespecified clinically relevant variables; a LASSO-selected model, including variables retained at $\lambda_{1\text{-SE}}$ in more than 40% of iterations; and a maximally

adjusted model, including the union of prespecified variables and LASSO-selected variables retained at λ_{min} in more than 40% of iterations. All models included delineation method as the exposure variable.

3.3.3 PSM-matched cohort analysis: To mitigate baseline imbalance, a propensity score–matched (PSM) cohort analysis was conducted. Covariates for propensity score estimation were selected a priori based on their clinical relevance to treatment allocation and surgical complexity. A parsimonious set of preoperative variables was deliberately adopted to avoid overfitting, given the limited number of HFUS+CEUS patients. Age, primary site, maximum tumor diameter, ultrasound morphology, deep fascia abutment, and ultrasound depth were included, as these variables capture key dimensions of tumor complexity and preoperative decision-making. Patients were matched using nearest-neighbor matching without replacement, with a caliper width of 0.2 standard deviations of the logit of the propensity score and a maximum matching ratio of 1:2. Covariate balance before and after matching was assessed using standardized mean differences.

In the PSM-matched cohort, categorical outcomes were compared using Cochran–Mantel–Haenszel tests stratified by matched subclasses, and continuous outcomes were compared using the van Elteren test, implemented as a stratified Wilcoxon rank-sum test. Multivariable Poisson regression models were fitted in the matched sample using matching weights, with inference based on cluster-robust standard errors clustered on matched subclasses.

3.3.4 Alternative-model analyses: To assess robustness to alternative modeling assumptions regarding the scale and distribution of the outcome, the primary outcome was alternatively specified as: (1) an ordinal outcome coded as 0, 1, 2, … extra peripheral Mohs stages and analyzed using ordinal logistic regression; (2) a binary outcome coded as 0 versus ≥1 extra peripheral Mohs stages and analyzed using binary logistic regression with logit link; and (3) a continuous outcome analyzed using linear regression.

In the unmatched cohort, inference for logistic and linear regression models was based on Wald tests using heteroskedasticity-robust variance estimators, and 95% confidence intervals were derived from the corresponding robust standard errors. For ordinal logistic regression in the unmatched cohort, odds ratios were estimated from proportional odds models, with confidence intervals obtained from the fitted model and P values from Wald-type tests. In the propensity score–matched cohort, regression models were fitted in the matched sample using matching weights, and inference was based on cluster-robust Wald tests and 95% confidence intervals clustered on matched subclasses.

3.4 Risk stratification analysis: To explore potential heterogeneity in the effect of delineation method, a risk-stratified analysis was performed based on predicted

baseline risk of requiring extra peripheral Mohs stages. A baseline risk model was developed in the HFUS-only group, with peripheral margin positivity as the outcome. Candidate predictors included baseline covariates retained in the primary multivariable analysis, excluding delineation method. The model was specified using binary logistic regression, and predicted probabilities were calculated for all patients in the full cohort. Patients were then stratified into tertiles (low, intermediate, and high risk) using cutoffs derived from the distribution of predicted risk in the HFUS-only group. Within each risk stratum, the association between delineation method (HFUS+CEUS vs HFUS-only) and extra peripheral Mohs stages was evaluated using Poisson regression models, and incidence rate ratios (IRRs) with 95% confidence intervals (CIs) were reported. To assess effect modification, an interaction term between delineation method and risk strata was included in the Poisson regression model fitted in the full cohort. Peripheral margin positivity rates within each stratum were additionally summarized for descriptive comparison.

3.5 Exploratory subgroup and interaction analyses: Exploratory subgroup analyses were conducted using multivariable Poisson regression. To facilitate clinical interpretability and ensure sufficient sample sizes for estimation within subgroups, continuous variables were dichotomized at their respective medians when defining these subgroups. For subgroups with few events, univariable Poisson regression was conducted. For subgroups with quasi-complete separation, bias-reduced Poisson regression using brglmFit was applied. Conversely, to prevent information loss and maximize statistical power during formal interaction testing, the original continuous forms of these variables were retained. Prespecified interaction terms between delineation method and selected clinicopathologic variables were evaluated in multivariable Poisson regression models. These analyses were considered exploratory and interpreted with caution.

3.6 Other assessments:

3.6.1 Dispersion assessment: Overdispersion was assessed by the ratio of residual deviance to residual degrees of freedom.

3.6.2 Zero-inflation assessment: Zero inflation was assessed to determine whether zero-inflated Poisson models were warranted.

3.6.3 Multicollinearity assessment: Variance inflation factors were used to assess multicollinearity among covariates in multivariable models.

3.6.4 Ordinal model diagnostics: The proportional odds assumption was evaluated to assess the appropriateness of the ordinal logistic regression model.

3.6.5 Calendar-time analyses: To explore potential temporal trends and assess possible learning-curve–related effects that may introduce time-related confounding, univariable Poisson regression analyses were conducted with calendar time as the independent variable and extra peripheral and deep Mohs stages as outcomes.

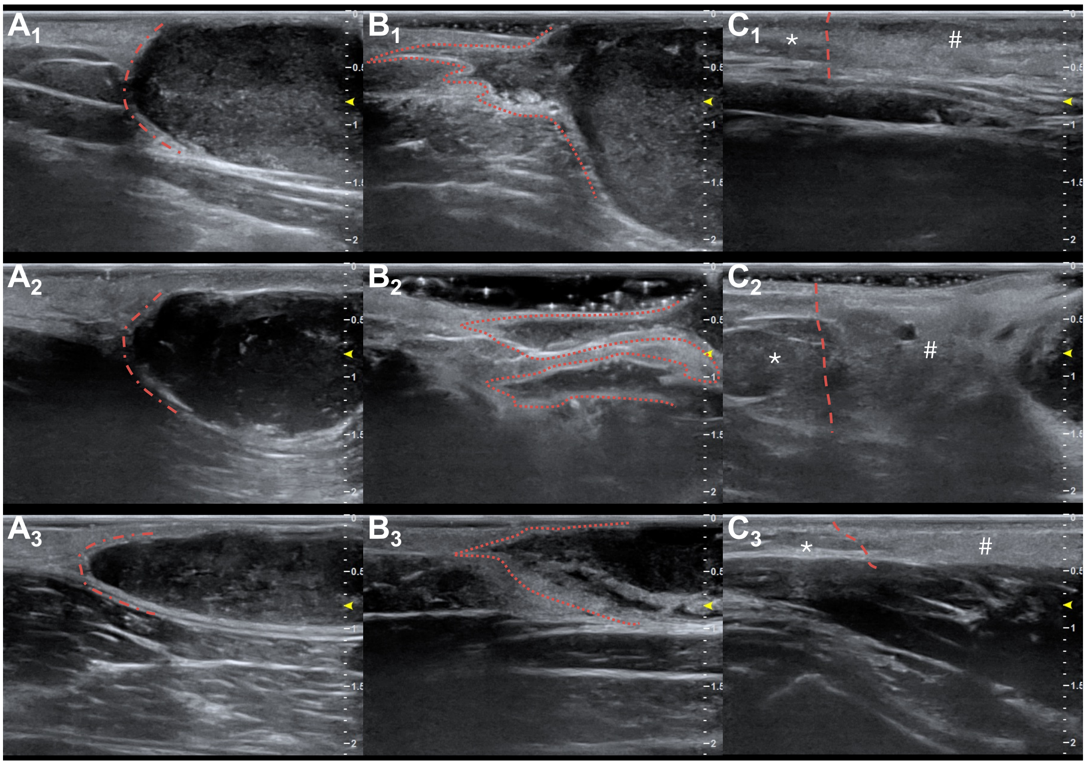

Supplementary Figure S1. Three representative HFUS margin patterns of DFSP. **(A_1–A_3)** Well-defined margins that can be readily identified on HFUS. The dash-dot line indicates the tumor boundary. This appearance is consistent with an expansive growth pattern. **(B_1–B_3)** An ill-defined margin with hypoechoic "tentacle-like" extensions, which can usually be recognized on HFUS by experienced sonographers. This appearance is consistent with an infiltrative growth pattern. The dotted line indicates the tumor boundary. **(C_1–C_3)** Another type of ill-defined margin, characterized by mixed echogenicity and subtle infiltration, in which the tumor boundary is difficult to identify on HFUS. The dashed line indicates the presumed tumor boundary, inferred from subtle grayscale differences and disruption of normal fibrous septa by experienced sonographers. All images were acquired using 16-MHz HFUS.

*, normal side; #, tumor side.

DFSP, dermatofibrosarcoma protuberans; HFUS, high-frequency ultrasound.

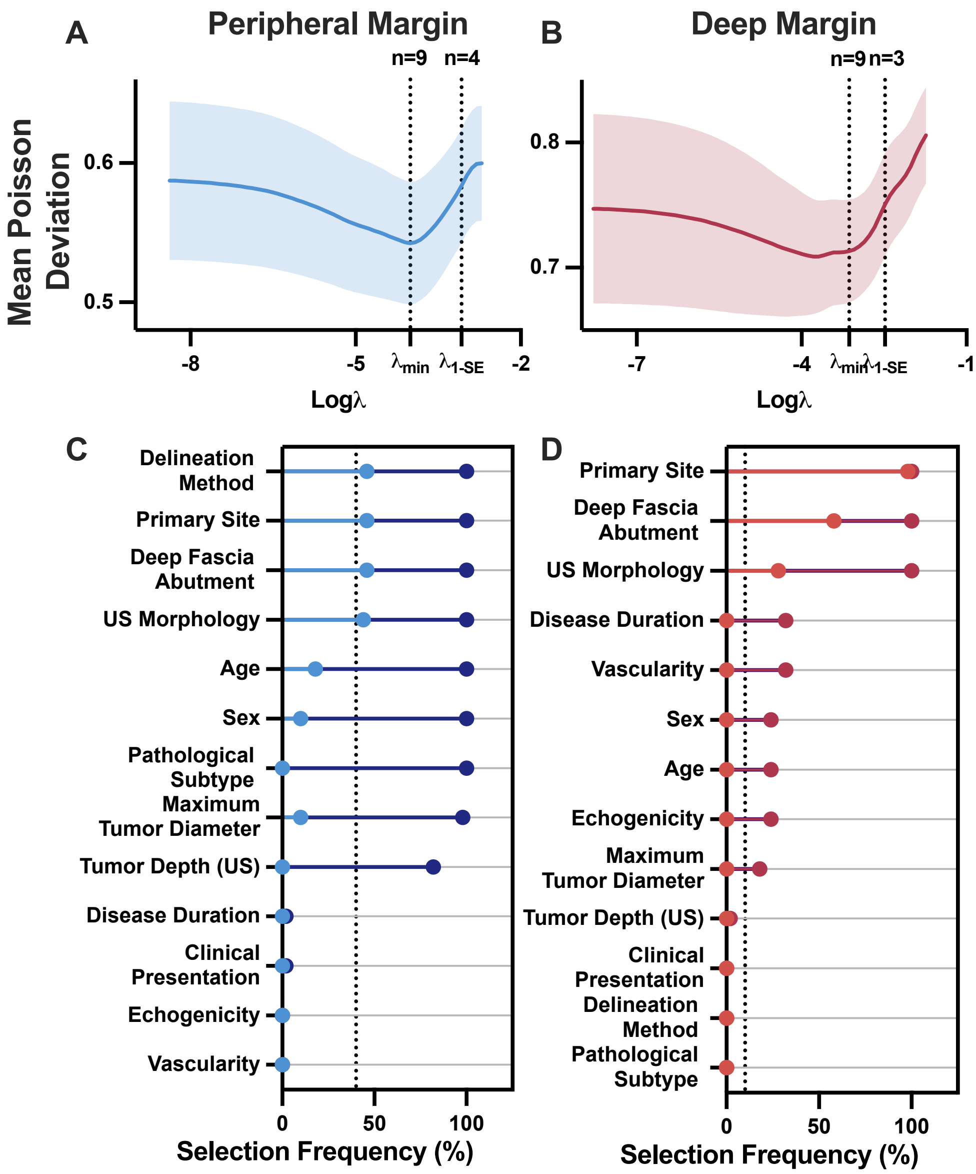

Supplementary Figure S2. Cross-validation curves and variable selection frequencies from repeated LASSO analyses for extra peripheral (panels A and C) and extra deep (panels B and D) Mohs stages. Panels **A** and **B** show representative cross-validation curves from a single LASSO Poisson regression fit, illustrating the mean Poisson deviance across values of the tuning parameter (λ). Dotted vertical lines indicate λ_{min} and $\lambda_{1\text{-SE}}$, and numbers above the lines indicate the number of non-zero coefficients at the corresponding λ values. These curves are shown for illustrative purposes and were not directly used for variable selection. Panels **C** and **D** show the selection frequency of each variable across repeated LASSO analyses (50 repetitions with 10-fold cross-validation). Variable selection was based on predefined frequency thresholds: for extra peripheral Mohs stages, variables selected in >40% of repetitions at $\lambda_{1\text{-SE}}$ were retained for multivariable modeling; for extra deep Mohs stages,

variables selected in >10% of repetitions at $\lambda_{1\text{-SE}}$ were retained.
LASSO, least absolute shrinkage and selection operator; US, ultrasound.

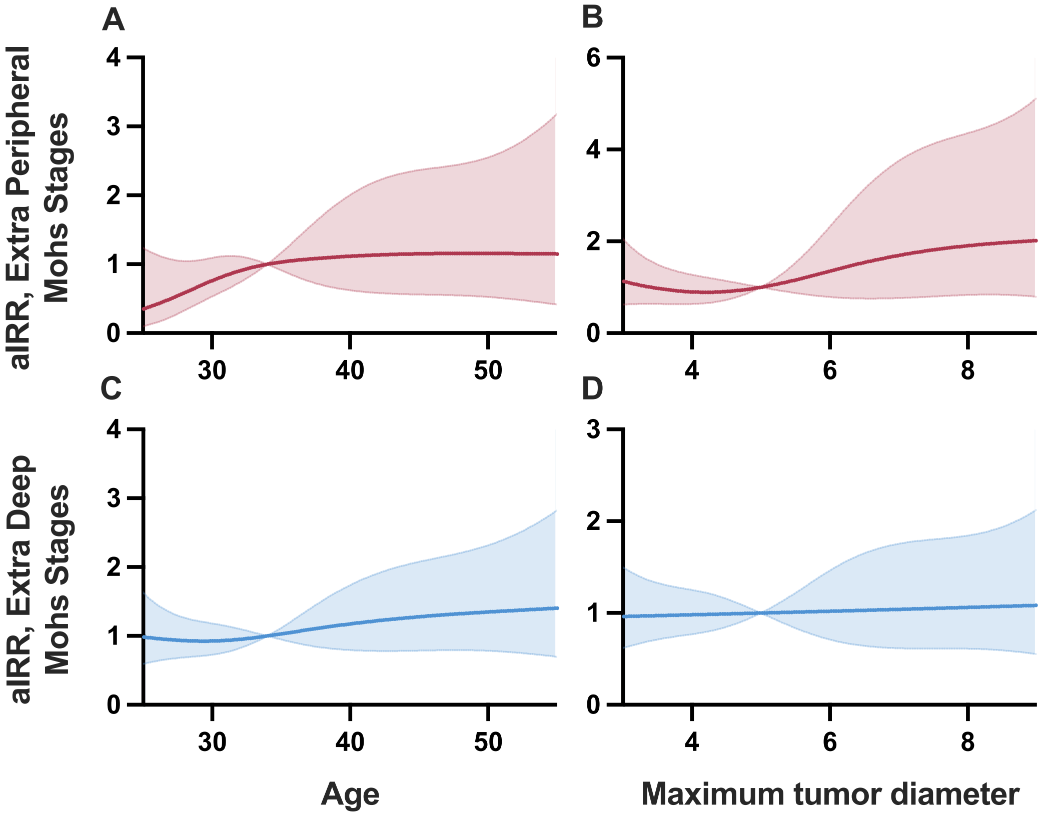

Supplementary Figure S3. Restricted cubic spline analyses of age and maximum tumor diameter in relation to extra peripheral and deep Mohs stages. Adjusted incidence rate ratios (aIRRs) and 95% confidence intervals (CIs) for extra peripheral Mohs stages **(A–B)** and extra deep Mohs stages **(C–D)** are shown as functions of age **(A, C)** and maximum tumor diameter **(B, D)**. Estimates were derived from multivariable Poisson regression models incorporating restricted cubic splines, adjusted for delineation method, primary site, the alternate continuous variable (age or tumor size), US morphology, and deep fascia abutment. Solid lines represent point estimates, and shaded areas indicate 95% confidence intervals. Reference values correspond to the median of each continuous variable.

aIRR, adjusted incidence rate ratio; CI, confidence interval.

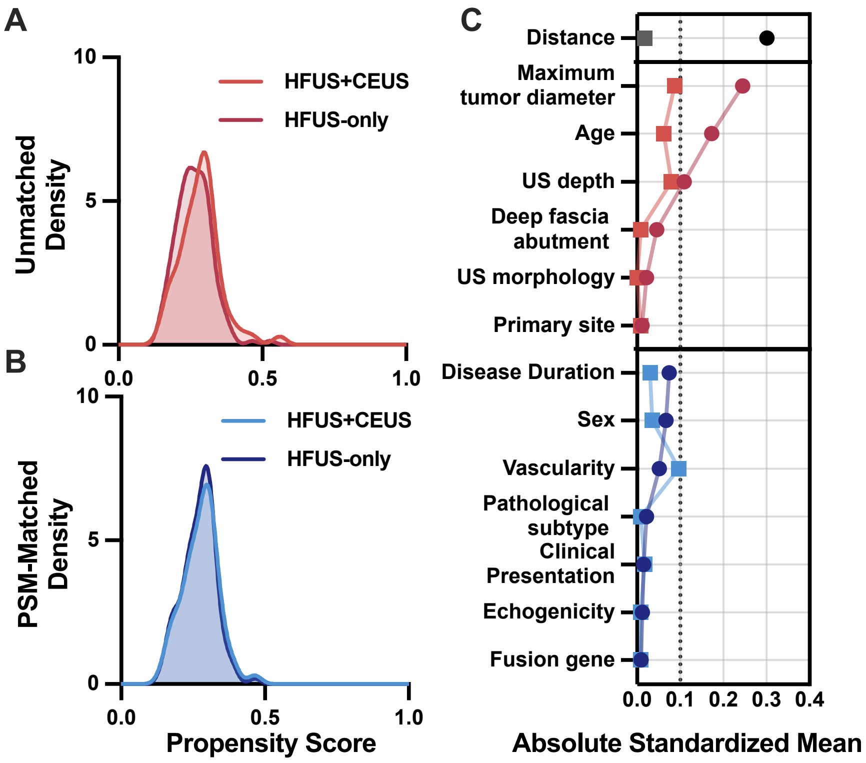

Supplementary Figure S4. Propensity score distribution and covariate balance before and after propensity score matching (PSM). (A) Distribution of propensity scores in the unmatched cohort. **(B)** Distribution of propensity scores in the PSM-matched cohort, demonstrating improved overlap between groups after matching. **(C)** Love plot showing absolute standardized mean differences (SMDs) for all covariates before (circles) and after (squares) matching. Variables are ordered according to their unmatched SMDs. Covariates used for propensity score estimation are displayed in the upper panel (red), whereas additional covariates not included in the propensity score model are shown in the lower panel (blue) for balance assessment. The vertical dashed line indicates an absolute SMD of 0.1, representing the conventional threshold for acceptable balance.

CEUS, contrast-enhanced ultrasound; HFUS, high-frequency ultrasound; PSM, propensity score matching; US, ultrasound.

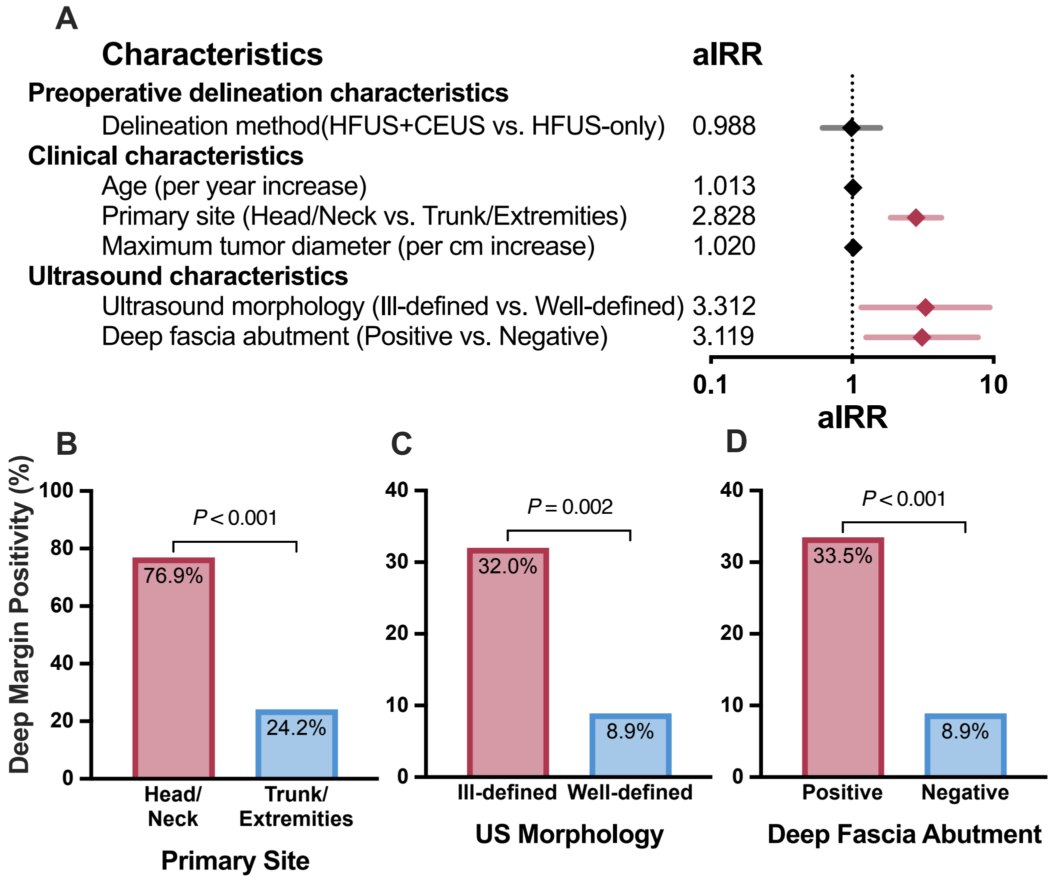

Supplementary Figure S5. Factors associated with extra deep Mohs stages and corresponding deep margin positivity. (A) Forest plot showing adjusted incidence rate ratios (aIRRs) and 95% confidence intervals (CIs) from the multivariable Poisson regression model. **(B–D)** Bar charts showing deep margin positivity rates across key strata, stratified by primary site **(B)**, US morphology **(C)**, and deep fascia abutment **(D)**. *P* values in panels **B–D** were calculated using the chi-square test. Deep margin positivity rates are presented for descriptive comparison.

aIRR, adjusted incidence rate ratio; CEUS, contrast-enhanced ultrasound; HFUS, high-frequency ultrasound; US, ultrasound.

Supplementary Table S1. CEUS characteristics of DFSP patients in the HFUS+CEUS group.

CEUS characteristics	HFUS+CEUS (n=59)
Arrival time, s, median (IQR)	13 (10, 15)
Enhancement intensity, No. (%)	
Marked hyperenhancement	53 (89.8)
Mild hyperenhancement	5 (8.5)
Isoenhancement	1 (1.7)
Hypoenhancement	0 (0.0)
Enhancement homogeneity, No. (%)	
Homogeneous enhancement	40 (67.8)
Heterogeneous enhancement	19 (32.2)

CEUS, contrast-enhanced ultrasound; DFSP, dermatofibrosarcoma protuberans; HFUS, high-frequency ultrasound.

The interquartile range (IQR) is presented as the 25th and 75th percentiles (Q1, Q3).

Supplementary Table S2. Univariable Poisson regression analysis of characteristics associated with extra peripheral Mohs stages in the entire cohort.

Characteristics	IRR	95% CI	*P* value
**Preoperative delineation characteristics**			
Delineation method (HFUS+CEUS vs. HFUS-only)	0.273	0.085 to 0.872	**0.028***
**Clinical characteristics**			
Age (per year increase)	1.025	1.001 to 1.049	**0.037***
Sex (Male vs. Female)	1.952	0.982 to 3.880	0.056
Primary site (Head/Neck vs. Trunk/Extremities)	2.843	1.297 to 6.236	**0.009***
Clinical presentation (Nodular/Subcutaneous vs. Plaque/Atrophic)	1.037	0.489 to 1.901	0.916
Maximum tumor diameter (per cm increase)	1.164	0.989 to 1.369	0.067
Disease duration (per month increase)	1.001	0.997 to 1.004	0.682
**Ultrasound characteristics**			
Ultrasound morphology (Ill-defined vs. Well-defined)	3.986	0.979 to 16.228	0.054
Echogenicity (Hyperechoic or mixed vs. Hypoechoic)	0.970	0.488 to 1.925	0.930
Tumor depth (US) (per cm increase)	1.155	0.856 to 1.559	0.345
Deep fascia abutment (Positive vs. Negative)	5.293	1.293 to 21.668	**0.020***
Vascularity (Rich vs. Poor)	1.179	0.533 to 2.606	0.685
**Pathological characteristics**			
Pathological subtype (FS-DFSP vs. C-DFSP)	1.615	0.765 to 3.412	0.209

C-DFSP, classic DFSP; CEUS, contrast-enhanced ultrasound; CI, confidence interval; DFSP, dermatofibrosarcoma protuberans; FS-DFSP, fibrosarcomatous DFSP; HFUS, high-frequency ultrasound; IRR, incidence rate ratio; US, ultrasound.

*$P < 0.05$ was considered statistically significant.

Supplementary Table S3. Variable selection frequencies in repeated LASSO Poisson regression models for extra peripheral Mohs stages.

Characteristics	λ_{min} selection frequency (%)	$\lambda_{1\text{-}SE}$ selection frequency (%)
Delineation method	100	46
Primary site	100	46
Deep fascia abutment	100	46
Ultrasound morphology	100	44
Age	100	18
Sex	100	10
Pathological subtype	100	0
Maximum tumor diameter	98	10
Tumor depth (US)	82	0
Disease duration	2	0
Clinical presentation	2	0
Echogenicity	0	0
Vascularity	0	0

Selection frequency was defined as the percentage of 50 repeated LASSO fits in which the variable had a non-zero coefficient. In each repetition, 10-fold cross-validation was performed to determine λ_{min} and $\lambda_{1\text{-}SE}$. Variables selected in more than 40% of repetitions at $\lambda_{1\text{-}SE}$ were retained for the multivariable adjustment. Variables selected in more than 40% of repetitions at λ_{min} were retained for the maximally adjusted model.

LASSO, least absolute shrinkage and selection operator; SE, standard error of the cross-validated error estimate; US, ultrasound.

λ_{min} refers to the regularization parameter that gives the minimum mean cross-validated error.

$\lambda_{1\text{-}SE}$ refers to the largest value of λ for which the cross-validated error is within one standard error of the minimum.

Supplementary Table S4. Multivariable Poisson regression analysis of characteristics associated with extra peripheral Mohs stages in the entire cohort.

Characteristics	aIRR	95% CI	*P* value
**Preoperative delineation characteristics**			
Delineation method (HFUS+CEUS vs. HFUS-only)	0.310	0.097 to 0.996	**0.049***
**Clinical characteristics**			
Age (per year increase)	1.030	1.004 to 1.057	**0.025***
Primary site (Head/Neck vs. Trunk/Extremities)	1.855	0.812 to 4.237	0.142
Maximum tumor diameter (per cm increase)	1.083	0.930 to 1.263	0.305
**Ultrasound characteristics**			
US morphology (Ill-defined vs. Well-defined)	4.135	0.997 to 17.141	0.050
Deep fascia abutment (Positive vs. Negative)	4.420	1.040 to 18.775	**0.044***

CEUS, contrast-enhanced ultrasound; CI, confidence interval; HFUS, high-frequency ultrasound; aIRR, adjusted incidence rate ratio; US, ultrasound.

*P < 0.05 was considered statistically significant.

Supplementary Table S5. Univariable Poisson regression analysis of calendar-time variables associated with extra peripheral and extra deep Mohs stages in the HFUS-only group and HFUS+CEUS group.

Calendar-time variables	IRR	95% CI	*P* value
**HFUS-only group**			
**Extra peripheral Mohs stages**			
per quarter	0.967	0.900 to 1.039	0.363
per 6-month interval	0.940	0.813 to 1.087	0.400
per year	0.850	0.627 to 1.150	0.290
2023–2024 vs. 2021–2022	0.769	0.373 to 1.586	0.473
**Extra deep Mohs stages**			
per quarter	1.017	0.962 to 1.077	0.556
per 6-month interval	1.035	0.925 to 1.164	0.549
per year	1.063	0.841 to 1.355	0.612
2023–2024 vs. 2021–2022	1.368	0.777 to 2.485	0.282
**HFUS+CEUS group**			
**Extra peripheral Mohs stages**			
per 10 patients	1.382	0.694 to 3.300	0.364
**Extra deep Mohs stages**			
per 10 patients	1.015	0.757 to 1.361	0.920

Time variables were modeled separately as continuous time scales (per quarter, per 6-month interval, and per year) and as a binary period indicator (2023–2024 vs. 2021–2022) for the HFUS-only group.

Because CEUS implementation was intermittent due to equipment availability, calendar-time–based analyses may not adequately capture potential learning effects. Therefore, a case-order metric (per 10 patients) was additionally used to explore potential learning-curve–related trends.

IRRs, 95% confidence intervals, and P values were obtained from univariable Poisson regression models with robust standard errors.

CI, confidence interval; CEUS, contrast-enhanced ultrasound; HFUS, high-frequency ultrasound; IRR, incidence rate ratio.

Supplementary Table S6. Sensitivity analyses of the association between delineation method and extra peripheral Mohs stages under alternative cohort restrictions and calendar-time adjustments.

Cohorts	Patients, n (HFUS-only/HFUS+CEUS)	Events, n	aIRR	95% CI	*P* value
Entire cohort	161/59	33	0.310	0.097 to 0.996	**0.049***
Entire cohort excluding 8 clinically ineligible cases	153/59	33	0.300	0.094 to 0.961	**0.043***
Entire cohort excluding 29 HFUS-only cases from CEUS-available periods	132/59	28	0.303	0.093 to 0.984	**0.047***
Contemporary cohort	65/59	13	0.283	0.081 to 0.986	**0.048***
Contemporary cohort, additionally adjusted for calendar time (per 6-month)	65/59	13	0.226	0.056 to 0.903	**0.035***
Contemporary cohort excluding 8 clinically ineligible cases	57/59	13	0.251	0.071 to 0.881	**0.031***
Strictly contemporary cohort	29/59	9	0.261	0.072 to 0.938	**0.040***
Strictly contemporary cohort excluding 8 clinically ineligible cases	21/59	9	0.198	0.056 to 0.700	**0.012***

"Clinically ineligible cases" refers to the 8 patients whose lesions were judged too small for CEUS and underwent HFUS when CEUS was available.

"HFUS-only cases from CEUS-available periods" refers to all 29 patients who underwent HFUS when CEUS was available.

"Contemporary cohort" refers to patients selected from March 1, 2023 to September 20, 2024.

"Strictly contemporary cohort" refers to patients selected from March 1, 2023 to June 30, 2023; August 16, 2023 to April 15, 2024; and August 19, 2024 to September 20, 2024.

For calendar-time adjustment, a per-6-month time variable (modeled as a continuous variable) was additionally included in the multivariable Poisson regression model together with delineation method, age, primary site, maximum tumor diameter, US morphology, and deep fascia abutment.

aIRR, adjusted incidence rate ratio; CEUS, contrast-enhanced ultrasound; CI, confidence interval; HFUS, high-frequency ultrasound.

*$P < 0.05$ was considered statistically significant.

Supplementary Table S7. Baseline comparability between the HFUS+CEUS group and alternative HFUS-only comparison groups.

Characteristics	HFUS+CEUS (n=59)	HFUS-only, entire cohort (n=161)	SMD	HFUS-only, contemporary cohort (n=65)	SMD	HFUS-only, strictly contemporary cohort (n=29)	SMD
**Clinical characteristics**							
Age, years, mean (SD)	36.7 (11.8)	34.6 (9.1)	0.193	34.0 (8.8)	0.246	33.0 (8.4)	0.356
Sex, No. (%)			0.067		0.003		0.094
Female	34 (57.6)	82 (50.9)		38 (58.5)		14 (48.3)	
Male	25 (42.4)	79 (49.1)		27 (41.5)		15 (51.7)	
Primary site, No. (%)			0.011		0.018		0.016
Head and neck	3 (5.1)	10 (6.2)		3 (4.6)		1 (3.4)	
Trunk and extremities	56 (94.9)	151 (93.8)		62 (95.4)		28 (96.6)	
Clinical presentation, No. (%)			0.015		0.001		0.008
Nodular/Subcutaneous	31 (52.5)	87 (54.0)		34 (52.3)		15 (51.7)	
Plaque/Atrophic	28 (47.5)	74 (46.0)		31 (47.7)		14 (48.3)	
Disease duration, months, median (IQR)	60 (12, 120)	48 (23, 120)	0.072	48 (12, 120)	0.008	24 (12, 120)	0.042
Maximum tumor diameter, cm, median (IQR)	4.8 (3.0, 6.1)	5.5 (3.9, 7.0)	0.234	4.5 (3.5, 7.5)	0.090	4.5 (3.0, 8.0)	0.123
**Ultrasound characteristics**							
Ultrasound morphology, No. (%)			0.022		0.009		0.048
Well-defined	13 (22.0)	32 (19.9)		15 (23.1)		5 (17.2)	
Ill-defined	46 (78.0)	129 (80.1)		50 (76.9)		24 (82.8)	

Characteristics	HFUS+CEUS (n=59)	HFUS-only, entire cohort (n=161)	SMD	HFUS-only, contemporary cohort (n=65)	SMD	HFUS-only, strictly contemporary cohort (n=29)	SMD
Echogenicity, No. (%)			0.012		0.031		0.077
Hyperechoic or mixed	26 (44.1)	69 (42.9)		27 (41.5)		15 (51.7)	
Hypoechoic	33 (55.9)	92 (57.1)		38 (58.5)		14 (48.3)	
Tumor depth (US), cm, median (IQR)	1.13 (0.84, 1.83)	1.21 (0.73, 1.91)	0.094	1.19 (0.73, 1.98)	0.104	1.02 (0.59, 1.95)	0.001
Deep fascia abutment, No. (%)			0.046		0.007		0.012
Negative	17 (28.8)	39 (24.2)		19 (29.2)		8 (27.6)	
Positive	42 (71.2)	122 (75.8)		46 (70.8)		21 (72.4)	
Vascularity, No. (%)			0.051		0.023		0.038
Poor (Adler 0-1)	12 (20.3)	41 (25.5)		13 (20.0)		7 (24.1)	
Rich (Adler 2-3)	47 (79.7)	120 (74.5)		52 (80.0)		22 (75.9)	
**Pathological characteristics**							
Pathological subtype, No. (%)			0.022		0.007		0.117
C-DFSP	46 (78.0)	122 (75.8)		51 (78.5)		26 (89.7)	
FS-DFSP	13 (22.0)	39 (24.2)		14 (21.5)		3 (10.3)	
COL1A1-PDGFB fusion gene, No. (%)			0.010		0.034		0.034
Positive	57 (96.6)	154 (95.7)		65 (100.0)		29 (100.0)	
Negative	2 (3.4)	7 (4.3)		0 (0.0)		0 (0.0)	

To assess the robustness of baseline comparability under progressively stricter calendar-time restrictions, the same HFUS+CEUS group was compared against three alternative HFUS-only groups derived from the entire, contemporary, and strictly contemporary cohorts.

Adler, a semi-quantitative classification of color Doppler blood flow; C-DFSP, classic DFSP; CEUS, contrast-enhanced ultrasound; COL1A1, collagen type I alpha 1 chain; DFSP,

dermatofibrosarcoma protuberans; FS-DFSP, fibrosarcomatous DFSP; HFUS, high-frequency ultrasound; IQR, interquartile range; PDGFB, platelet-derived growth factor beta chain; SD, standard deviation; SMD, standardized mean difference; US, ultrasound.

The interquartile range (IQR) is presented as the 25th and 75th percentiles (Q1, Q3).

Supplementary Table S8. Sensitivity analyses of the association between delineation method and extra peripheral Mohs stages across alternative covariate specifications and outcome-modeling frameworks.

Models	Variables	Patients, n (HFUS-only/HFUS+CEUS)	Effect estimates, aIRR/aOR/β	95% CI	*P* value
**Multivariable models in the entire cohort:**					
Primary Poisson model	6	161/59	aIRR 0.310	0.097 to 0.996	**0.049***
Prespecified model	4	161/59	aIRR 0.268	0.083 to 0.863	**0.027***
LASSO-selected model	4	161/59	aIRR 0.308	0.098 to 0.969	**0.044***
Maximally adjusted model	9	161/59	aIRR 0.297	0.097 to 0.910	**0.034***
Ordinal logistic model	6	161/59	aOR 0.259	0.059 to 0.806	**0.037***
Binary logistic model	6	161/59	aOR 0.259	0.070 to 0.958	**0.043***
Linear regression model	6	161/59	β -0.128	-0.212 to -0.043	**0.003***
**Multivariable models in the PSM-matched cohort:**					
Primary Poisson model	6	110/57	aIRR 0.309	0.105 to 0.909	**0.033***
Prespecified model	4	110/57	aIRR 0.298	0.104 to 0.849	**0.024***
LASSO-selected model	4	110/57	aIRR 0.297	0.101 to 0.879	**0.028***
Maximally adjusted model	9	110/57	aIRR 0.298	0.102 to 0.870	**0.027***
Ordinal logistic model	6	110/57	aOR 0.260	0.080 to 0.850	**0.026***
Binary logistic model	6	110/57	aOR 0.262	0.080 to 0.851	**0.026***
Linear regression model	6	110/57	β -0.127	-0.213 to -0.042	**0.004***

The prespecified Poisson model included the delineation method and the 3 clinically relevant variables.

The LASSO-selected Poisson model included variables selected using the $\lambda_{1\text{-SE}}$ rule in LASSO regression.

The maximally adjusted Poisson model included the union of prespecified and LASSO-selected variables by λ_{min} rule.

For ordinal logistic regression, extra peripheral Mohs stages were modeled as an ordinal outcome with ordered levels (0, 1, 2, ...).

For binary logistic regression, extra peripheral Mohs stages were modeled as a binary outcome (0 vs ≥1).

For linear regression analysis, extra peripheral Mohs stages were additionally treated as a continuous variable.

aIRR, adjusted incidence rate ratio; aOR, adjusted odds ratio; β, regression coefficient from linear model; CI, confidence interval; CEUS, contrast-enhanced ultrasound; HFUS, high-frequency ultrasound; PSM, propensity score matching.

*$P < 0.05$ was considered statistically significant.

Supplementary Table S9. Baseline clinical and tumor characteristics in the propensity score–matched cohort by delineation method (HFUS-only vs HFUS+CEUS).

Characteristics	Matched cohort (n = 167)	HFUS-only (n = 110)	HFUS+CEUS (n = 57)	SMD
**Clinical characteristics**				
Age, years, mean (SD)	35.9 (9.4)	36.0 (9.2)	35.6 (9.9)	0.062
Sex, No. (%)				0.035
Female	94 (56.3)	60 (54.5)	34 (59.6)	
Male	73 (43.7)	50 (45.5)	23 (40.4)	
Primary site, No. (%)				0.009
Head and neck	10 (6.0)	7 (6.4)	3 (5.3)	
Trunk and extremities	157 (94.0)	103 (93.6)	54 (94.7)	
Clinical presentation, No. (%)				0.018
Nodular/Subcutaneous	83 (49.7)	55 (50.0)	28 (49.1)	
Plaque/Atrophic	84 (50.3)	55 (50.0)	29 (50.9)	
Disease duration, months, median (IQR)	48 (12, 120)	36 (12, 120)	60 (24, 120)	0.030
Maximum tumor diameter, cm, median (IQR)	5.0 (3.5, 6.6)	5.0 (3.6, 7.0)	5.0 (3.2, 6.1)	0.087
**Ultrasound characteristics**				
Ultrasound morphology, No. (%)				0.000
Well-defined	32 (19.2)	21 (19.1)	11 (19.3)	
Ill-defined	135 (80.8)	89 (80.9)	46 (80.7)	
Echogenicity, No. (%)				0.009

Characteristics	Matched cohort (n = 167)	HFUS-only (n = 110)	HFUS+CEUS (n = 57)	SMD
Hyperechoic or mixed	77 (46.1)	51 (46.4)	26 (45.6)	
Hypoechoic	90 (53.9)	59 (53.6)	31 (54.4)	
Tumor depth (US), cm, median (IQR)	1.20 (0.73, 1.86)	1.22 (0.68, 1.90)	1.13 (0.84, 1.82)	0.080
Deep fascia abutment, No. (%)				0.009
Negative	47 (28.1)	31 (28.2)	16 (28.1)	
Positive	120 (71.9)	79 (71.8)	41 (71.9)	
Vascularity, No. (%)				0.097
Poor (Adler 0-1)	46 (27.5)	34 (30.9)	12 (21.1)	
Rich (Adler 2-3)	121 (72.5)	76 (69.1)	45 (78.9)	
**Pathological characteristics**				
Pathological subtype, No. (%)				0.009
C-DFSP	131 (78.4)	86 (78.2)	45 (78.9)	
FS-DFSP	36 (21.6)	24 (21.8)	12 (21.1)	
COL1A1-PDGFB fusion gene, No. (%)				0.009
Positive	160 (95.8)	105 (95.5)	55 (96.5)	
Negative	7 (4.2)	5 (4.5)	2 (3.5)	

Adler, a semi-quantitative classification of color Doppler blood flow; C-DFSP, classic DFSP; CEUS, contrast-enhanced ultrasound; COL1A1, collagen type I alpha 1 chain; DFSP, dermatofibrosarcoma protuberans; FS-DFSP, fibrosarcomatous DFSP; HFUS, high-frequency ultrasound; IQR, interquartile range; PSM, propensity score matching; PDGFB, platelet-derived growth factor beta chain; SD, standard deviation; SMD, standardized mean difference; US, ultrasound.

The interquartile range (IQR) is presented as the 25th and 75th percentiles (Q1, Q3).

Supplementary Table S10. Mohs surgical outcomes in the propensity score–matched cohort by delineation method (HFUS-only vs HFUS+CEUS).

Characteristics	Matched cohort (n = 167)	HFUS-only (n = 110)	HFUS+CEUS (n = 57)	*P* value
Peripheral Mohs stages, Mean (SD)	1.14 (0.37)	1.19 (0.42)	1.05 (0.23)	**0.016***†
Peripheral margin status, No. (%)				
Negative	144 (86.2)	90 (81.8)	54 (94.7)	**0.032***‡
Positive	23 (13.8)	20 (18.2)	3 (5.3)	
Deep Mohs stages, Mean (SD)	1.31 (0.53)	1.33 (0.56)	1.28 (0.45)	0.957†
Deep margin status, No. (%)				
Negative	120 (71.9)	79 (71.8)	41 (71.9)	0.901‡
Positive	47 (28.1)	31 (28.2)	16 (28.1)	
Total Mohs stages, Mean (SD)	1.37 (0.55)	1.39 (0.59)	1.31 (0.47)	0.722†
Total margin status, No. (%)				
Negative	112 (67.1)	73 (66.4)	39 (68.4)	0.902‡
Positive	55 (32.9)	37 (33.6)	18 (31.6)	
Reconstruction method, No. (%)				0.665‡
Primary closure/local flap	161 (96.4)	105 (95.5)	56 (98.2)	
Skin graft	6 (3.6)	5 (4.5)	1 (1.8)	
Deepest plane of resection, No. (%)				0.999‡
Deep fascia	110 (65.9)	72 (65.5)	38 (66.7)	
Subfascial tissue (muscle/bone)	57 (34.1)	38 (34.5)	19 (33.3)	

Total Mohs stages were defined as the maximum of peripheral and deep stages.

Peripheral, deep, and total Mohs stages were compared using the van Elteren test, as these variables are not normally distributed.

Peripheral, deep, and total margin positivity were compared using the Cochran-Mantel-Haenszel (CMH) test.

Mean (SD) values are presented for descriptive comparison with previous studies.

CEUS, contrast-enhanced ultrasound; DFSP, dermatofibrosarcoma protuberans; HFUS, high-frequency ultrasound; PSM, propensity score matching; SD, standard deviation.

*Differences were considered statistically significant at $P < 0.05$.

†van Elteren test; ‡Cochran-Mantel-Haenszel (CMH) test.

Supplementary Table S11. Distribution of peripheral margin positivity and extra peripheral Mohs stages across risk strata in the HFUS-only cohort.

Risk Stratification	Patients, n	Peripheral margin positivity	Extra peripheral Mohs stages
Low risk	54	1	1
Intermediate risk	53	6	6
High risk	54	21	23

Risk strata were defined based on tertiles of predicted risk derived from the HFUS-only group to avoid potential bias from incorporating treatment-related information (i.e., CEUS use) into the stratification process and to preserve the baseline risk structure independent of the exposure.

HFUS, high-frequency ultrasound.

Supplementary Table S12. Stratified association between delineation method and extra peripheral Mohs stages across baseline risk strata.

Risk Stratification	Patients, n (HFUS-only/HFUS+CEUS)	Events, n	Stratified IRR	95% CI	*P* value
Low risk	54/20	2	2.700	0.169 to 43.168	0.482
Intermediate risk	53/20	7	0.442	0.053 to 3.669	0.449
High risk	54/19	24	0.124	0.017 to 0.915	**0.041***

P for interaction = 0.077.

Incidence rate ratios (IRRs) and 95% confidence intervals (CIs) were estimated using Poisson regression models within each stratum, comparing HFUS+CEUS with HFUS-only. *P* for interaction was calculated from a model including an interaction term between delineation method and risk strata in the full cohort.

CEUS, contrast-enhanced ultrasound; CI, confidence interval; HFUS, high-frequency ultrasound; IRR, incidence rate ratio.

*$P < 0.05$ was considered statistically significant.

Supplementary Table S13. Subgroup analyses of the association between delineation method and extra peripheral Mohs stages in the entire cohort.

Subgroup	Patients, n (HFUS-only/HFUS+CEUS)	Events, n	IRR/aIRR	95% CI	*P* value	*P* value for interaction[a]
Primary Site						0.201
Head/Neck[b]	10/3	5	IRR 0.833	0.043 to 5.633	0.868	
Trunk/Extremities	151/56	28	aIRR 0.225	0.053 to 0.950	**0.042***	
US morphology						0.992
Well-defined[c]	32/13	2	IRR 0.492	0.143 to 1.689	0.260	
Ill-defined	129/46	31	aIRR 0.355	0.111 to 1.134	0.080	
Deep fascia abutment						0.148
Positive	122/42	31	aIRR 0.218	0.053 to 0.902	**0.036***	
Negative[b]	39/17	2	IRR 2.294	0.091 to 58.014	0.563	
Age (years)						0.812
≤ 34	88/30	15	aIRR 0.410	0.101 to 1.665	0.212	
> 34	73/29	18	aIRR 0.208	0.024 to 1.803	0.154	
Maximum tumor diameter (cm)						0.426
≤ 5.0[d]	76/35	11	aIRR 0.366	0.111 to 1.209	0.099	
> 5.0	85/24	22	aIRR 0.367	0.090 to 1.503	0.164	

Subgroup-specific associations between delineation method and extra peripheral Mohs stages were assessed using subgroup-specific Poisson regression models. Standard univariable Poisson regression was used unless otherwise specified.

a: P for interaction was derived from interaction terms added separately to the multivariable Poisson regression model fitted in the entire cohort, rather than from the subgroup-specific models shown in this table.

b: Univariable Poisson regression models were used in these groups because of sparse events; these estimates should be interpreted cautiously.

c: A bias-reduced univariable Poisson regression model was used in the “well-defined” subgroup because of quasi-complete separation and a few events.

d: Bias-reduced multivariable Poisson regression model was used in this group because of quasi-complete separation.

Age and maximum tumor diameter were dichotomized at the median values of the entire cohort (34 years and 5.0 cm, respectively).

aIRR, adjusted incidence rate ratio; CEUS, contrast-enhanced ultrasound; CI, confidence interval; HFUS, high-frequency ultrasound; IRR, incidence rate ratio; US, ultrasound.

*$P < 0.05$ was considered statistically significant.

Supplementary Table S14. Pairwise associations among selected clinical, sonographic, and pathological characteristics.

Characteristics analyzed	Subgroup 1	Subgroup 2	*P* value
Maximum tumor diameter vs. Deep fascia abutment	≤ 5 cm (n = 111)	> 5 cm (n = 109)	**< 0.001***
Negative, No. (%)	45 (40.5)	11 (10.1)	
Positive, No. (%)	66 (59.5)	98 (89.9)	
Pathological subtype vs. Maximum tumor diameter	C-DFSP (n = 168)	FS-DFSP (n = 52)	**< 0.001***
≤ 5 cm, No. (%)	98 (58.3)	13 (25.0)	
> 5 cm, No. (%)	70 (41.7)	39 (75.0)	
Pathological subtype vs. Deep fascia abutment	C-DFSP (n = 168)	FS-DFSP (n = 52)	**< 0.001***
Negative, No. (%)	52 (31.0)	4 (7.7)	
Positive, No. (%)	116 (69.0)	48 (92.3)	
Pathological subtype vs. US morphology	C-DFSP (n = 168)	FS-DFSP (n = 52)	**< 0.001***
Well-defined, No. (%)	23 (13.7)	22 (42.3)	
Ill-defined, No. (%)	145 (86.3)	30 (57.7)	

P values were derived from chi-square tests.

C-DFSP, classic DFSP; DFSP, dermatofibrosarcoma protuberans; FS-DFSP, fibrosarcomatous DFSP; US, ultrasound.

**P* < 0.05 was considered statistically significant.

Supplementary Table S15. Univariable Poisson regression analysis of characteristics associated with extra deep Mohs stages in the entire cohort.

Characteristics	IRR	95% CI	*P* value
**Preoperative delineation characteristics**			
Delineation method (HFUS+CEUS vs. HFUS-only)	0.873	0.530 to 1.438	0.594
**Clinical characteristics**			
Age (per year increase)	1.011	0.989 to 1.032	0.336
Sex (Male vs. Female)	1.423	0.898 to 2.256	0.134
Primary site (Head/Neck vs. Trunk/Extremities)	3.906	2.496 to 6.111	**<0.001***
Clinical presentation (Nodular/Subcutaneous vs. Plaque/Atrophic)	1.089	0.691 to 1.716	0.714
Maximum tumor diameter (per cm increase)	1.069	0.951 to 1.201	0.265
Disease duration (per month increase)	1.002	1.000 to 1.004	0.111
**Ultrasound characteristics**			
Ultrasound morphology (Ill-defined vs. Well-defined)	3.137	1.125 to 8.746	**0.029***
Echogenicity (Hyperechoic or mixed vs. Hypoechoic)	1.316	0.836 to 2.071	0.236
Tumor depth (US) (per cm increase)	0.835	0.577 to 1.206	0.336
Deep fascia abutment (Positive vs. Negative)	3.415	1.363 to 8.557	**0.009***
Vascularity (Rich vs. Poor)	0.730	0.449 to 1.185	0.203
**Pathological characteristics**			
Pathological subtype (FS-DFSP vs. C-DFSP)	0.870	0.499 to 1.515	0.622

C-DFSP, classic DFSP; CEUS, contrast-enhanced ultrasound; CI, confidence interval; DFSP, dermatofibrosarcoma protuberans; FS-DFSP, fibrosarcomatous DFSP; HFUS, high-frequency ultrasound; IRR, incidence rate ratio; US, ultrasound.

*$P < 0.05$ was considered statistically significant.

Supplementary Table S16. Variable selection frequencies in repeated LASSO Poisson regression models for extra deep Mohs stages.

Characteristics	λ_{min} selection frequency (%)	$\lambda_{1\text{-}SE}$ selection frequency (%)
Primary site	100	98
Deep fascia abutment	100	58
Ultrasound morphology	100	28
Disease duration	32	0
Vascularity	32	0
Sex	24	0
Age	24	0
Echogenicity	24	0
Maximum tumor diameter	18	0
Tumor depth (US)	2	0
Clinical presentation	0	0
Delineation method	0	0
Pathological subtype	0	0

Selection frequency was defined as the percentage of 50 repeated LASSO fits in which the variable had a non-zero coefficient. In each repetition, 10-fold cross-validation was performed to determine λ_{min} and $\lambda_{1\text{-}SE}$. Variables selected in more than 10% of repetitions at $\lambda_{1\text{-}SE}$ were retained for the multivariable adjustment.

LASSO, least absolute shrinkage and selection operator; SE, standard error of the cross-validated error estimate; US, ultrasound.

λ_{min} refers to the regularization parameter that gives the minimum mean cross-validated error.

$\lambda_{1\text{-}SE}$ refers to the largest value of λ for which the cross-validated error is within one standard error of the minimum.

Supplementary Table S17. Multivariable Poisson regression analysis of characteristics associated with extra deep Mohs stages in the entire cohort.

Characteristics	aIRR	95% CI	*P* value
**Preoperative delineation characteristics**			
Delineation method (HFUS+CEUS vs. HFUS-only)	0.988	0.617 to 1.582	0.962
**Clinical characteristics**			
Age (per year increase)	1.013	0.990 to 1.035	0.278
Primary site (Head/Neck vs. Trunk/Extremities)	2.828	1.866 to 4.287	**<0.001***
Maximum tumor diameter (per cm increase)	1.020	0.929 to 1.120	0.680
**Ultrasound characteristics**			
US morphology (Ill-defined vs. Well-defined)	3.312	1.162 to 9.438	**0.025***
Deep fascia abutment (Positive vs. Negative)	3.119	1.253 to 7.760	**0.014***

aIRR, adjusted incidence rate ratio; CEUS, contrast-enhanced ultrasound; CI, confidence interval; HFUS, high-frequency ultrasound; US, ultrasound.

*$P < 0.05$ was considered statistically significant.
